# Methods for assessing exercise fidelity in unsupervised home-based cardiovascular rehabilitation: a scoping review

**DOI:** 10.1186/s13102-025-01069-7

**Published:** 2025-02-28

**Authors:** Mohammad Jarallah, Thomas M Withers, Sheeba Rosewilliam, Afroditi Stathi, Colin J Greaves

**Affiliations:** 1 School of Sport, Exercise and Rehabilitation Sciences, University of Birmingham, Edgbaston, Birmingham, B15 2TT UK; 2 Department of Physical Therapy and Health Rehabilitation, College of Applied Medical Sciences, Majmaah University, Majmaah, 11952 Kingdom of Saudi Arabia

**Keywords:** Exercise Fidelity, Exercise Adherence, Home-based Exercise, Cardiac Rehabilitation, Cardiovascular diseases, Treatment adherence and compliance, Adherence interventions, Behavior and behavior mechanisms, Exercise Therapy, Exercise

## Abstract

**Background:**

Home-based cardiac rehabilitation is increasingly popular for patients with cardiovascular diseases. However, this mode of rehabilitation involves unsupervised exercise, making it challenging to assess, correct, and monitor exercise fidelity (the extent to which the patient performs the exercise intended by the intervention provider). This review aimed to identify the range, validity, and acceptability of measures for assessing exercise fidelity in unsupervised, home-based cardiovascular rehabilitation.

**Methods:**

We searched PubMed, Embase, CINAHL, Medline, and PsycINFO for studies published between 2000 and 2024 to identify observational studies, trials, and protocols published in English with a home-based cardiovascular rehabilitation intervention and at least one measure of exercise fidelity (e.g., adherence to the intended frequency, intensity, time, type, safety, progression/regression). Two reviewers selected eligible studies and extracted data, including study characteristics, exercise components, adherence definition, adherence measures, and data on measurement validity or acceptability. We conducted a narrative synthesis using a comprehensive definition of exercise fidelity, which evolved as the analysis progressed.

**Results:**

Forty-six articles describing 41 studies were included. Exercise intensity was the most commonly measured fidelity component (38/41 studies), followed by exercise frequency (32/41 studies). Exercise intensity was mostly assessed by wearable devices (28/41 studies). Frequency of exercise was most commonly assessed subjectively using a self-reported exercise log or diary, but also (objectively) using wearable devices. Exercise quantity was most commonly assessed (in terms of time or duration) by self-reported exercise logs, diaries and wearable devices, or (in terms of steps or distance) mostly by wearable devices (pedometers, other step activity monitors). Safety was only assessed in 12/41 studies. No studies assessed progression or regression of exercise, quality of exercise (accuracy of movement) or the appropriateness of progression or regression.

**Conclusions:**

Most studies to date have conceptualised exercise fidelity or adherence narrowly, ignoring important elements of the initial exercise prescription and many have relied on non-validated self-report measures. We present a comprehensive framework for assessing exercise fidelity, which may be useful for designing more robust assessments of exercise fidelity in home-based rehabilitation programmes.

**Supplementary Information:**

The online version contains supplementary material available at 10.1186/s13102-025-01069-7.

## Introduction

Cardiovascular disease affects people globally, and its prevalence is growing rapidly [1], with an increase in cardiovascular mortality of 41% between 1990 and 2013, despite a decline in age-specific mortality [2]. Cardiovascular diseases cause a substantial economic burden. For example, cardiovascular diseases cost the European Union approximately €169 billion per year in 2003, representing 62% of overall health costs [3].

Exercise-based cardiac rehabilitation (CR) is an effective treatment for a number of cardiovascular diseases that improve quality of life and reduce hospital readmissions and mortality [4, 5]. For example, there is strong evidence from randomised controlled trials and systematic reviews that adding exercise-based cardiac rehabilitation to usual care improves the health-related quality of life and exercise capacity of patients with heart failure, compared with usual care alone [4, 6]. Similarly, evidence-based clinical guidelines recommend cardiac rehabilitation for acute coronary syndrome, revascularisation and myocardial infarction [7, 8].

Home-based cardiac rehabilitation is an alternative mode of delivery of cardiac rehabilitation, which has been shown to have a similar effect to centre-based cardiac rehabilitation on health-related quality of life and clinical outcomes in patients with heart disease [9, 10]. However, evidence suggests that the effects of home-based rehabilitation on exercise capacity may be sub-optimal: For example, in controlled /supervised settings, exercise-based rehabilitation increases exercise capacity in patients with heart failure (HF) [6, 11], but in the largest clinical trial to date of home-based cardiac rehabilitation for people with HF, there was no significant effect on exercise capacity [12].

In centre-based cardiac rehabilitation, it is easy to assess, monitor, and correct the quality of exercise that patients undertake. However, in home-based cardiac rehabilitation, exercise is not supervised. Therefore, assessing and correcting the quality, quantity and intensity of exercise undertaken by the patient is more challenging [13]. Hence, it is plausible that the reduced effect on exercise capacity seen in real-world trials of home-based cardiac rehabilitation may be due to a lack of “exercise fidelity” (the extent to which the patient performs an exercise in the way intended by the intervention provider). Furthermore, there is a lack of clear guidance or criteria for measuring exercise adherence for home-based cardiac rehabilitation [14].

Adherence in health contexts is defined as *“the extent to which a person’s behaviour corresponds with agreed recommendations from a health care provider”* [15]. However, this broad definition needs further specification for the context of home-based exercise. Prior studies have used heterogeneous definitions of exercise adherence, with adherence being defined mostly in terms of attendance at exercise sessions or adherence to a prescribed intensity or duration of exercise. However, there is no agreed definition within the literature [16]. As an initial starting point, in this article, we have used the more specific term “exercise fidelity” to describe *“The degree of adherence to the prescribed exercise programme in terms of the prescribed Frequency*,* Intensity*,* Time*,* Type*/*quality of exercise (accuracy of the movement)*,* Safety procedures and Progression/Regression”*.This starting-point definition incorporates the recommended components of American College of Sports Medicine (ACSM) for the prescription of exercise: *“Frequency (how often)”*,* “Intensity (how hard)”*,* “Time (duration or how long)”*,* “Type (mode or what kind)”* and progression/regression (adjustment of intensity over time) [17], as well as a *“Safety”* component which the authors felt to be an important consideration, particularly in a cardiac rehabilitation context.

A 2017 systematic review of exercise adherence measures in home-based rehabilitation found that self-administered questionnaires and diaries were mostly used [14]. In addition, this systematic review found that “step watch activity monitors” could be valid for measuring adherence to walking, duration, and frequency of exercise. However, this systematic review was limited to randomised controlled trials (RCT) designs and musculoskeletal conditions. In addition, this review used a more limited definition of adherence (“*the extent to which individuals undertake a prescribed behaviour accurately and at the agreed frequency*,* intensity*,* and duration”)*, which does not take into account the issues of safety or exercise progression /regression. A 2014 systematic review of adherence measurement for unsupervised home rehabilitation focused only on self-reported measures of adherence (defined loosely as “*the degree behaviour corresponds with an agreed on recommendation”)* [13].

Therefore, the aims of this scoping review were (a) to identify the range of possible measures for assessing “exercise fidelity” (based on our multi-component definition) in unsupervised/home-based cardiovascular rehabilitation programmes (b) to summarise existing data on the validity, reliability, and acceptability of measures of assessing exercise fidelity in unsupervised home-based cardiovascular rehabilitation.

## Methods

This scoping review has been registered on the Open Science Framework (https://osf.io/45f6m) and is reported in line with the PRISMA guidance on reporting scoping reviews [18].

### Eligibility

The inclusion and exclusion criteria are presented in Table 1.

**Table 1:** Inclusion and exclusion criteria

	Inclusion	Exclusion
**Population**	Adults (age 18+) with cardiovascular diseases, including heart failure, myocardial infarction, stroke, aneurysm, AF, cardiomyopathy, coronary artery disease, heart attack, deep vein thrombosis, heart valve disease, pericardial disease, rheumatic disease, and postcardiac surgery any ethnicity any country any socioeconomic status	Healthy individuals Animal studies Obesity Hypertension Non-cardiovascular diseases Stroke Congenital heart disease Peripheral artery disease or Intermittent Claudication
**Context**	Home-based exercise programme with a specified exercise prescription (i.e., a set of exercises specified in terms of any of the following components: Frequency, Intensity, Type, or Time/duration Type /quality of exercise (accuracy of the movement), Safety procedures, Progression / Regression. Unsupervised home-based exercise	Centre-based exercise programme Directed /supervised exercises, Speech exercises, breathing devices. Interventions with more than 20% of sessions being supervised.
**Concept**	Studies using measures to assess any component of exercise fidelity (as defined in the Introduction). Data on usability /acceptability of measures of exercise fidelity. Definitions of exercise fidelity. Measures had to be taken throughout the intervention period or during sessions, not only at baseline and follow-up (i.e., measures taken to assess study outcomes).	Measures of total weekly or daily physical activity (not specific to an exercise session or component of the home-based exercise prescription).
**Study design**	Any quantitative study with an exercise intervention component, such as observational studies and controlled studies (non-randomised controlled and RCT) Protocols for the above study designs	Reviews Qualitative studiesCohort studies (studies predicting adherence)
**Other criteria**	English language Peer-reviewed publication Published protocol Published from 2000 to 2024	Conference abstracts or report PhD thesis

### Search strategy

We searched PubMed, Embase, CINAHL, MEDLine and PsycINFO bibliographic databases for articles published in English between January 2000 and September 2024.We assumed that unsupervised home-based CR exercise was not in widespread use before 2000 in clinical practice. Furthermore, the technologies for assessing exercise fidelity used before this date are likely to be almost entirely based on self-report [19]. As such, extending the date range prior to 2000 would not add much to the study findings. We did not have the resources or capacity to process papers in languages other than English. The detailed search strategy for Embase is provided in Additional File 1. We developed sets of search terms and MeSH headings representing the following conceptual clusters:

1. **Cluster 1: Exercise**. Terms representing the intervention of interest (exercise-based cardiac rehabilitation).
2. **Cluster 2: Measures of adherence.** Terms representing measures of exercise fidelity (based on our initial definition of exercise fidelity above).
3. **Cluster 3: Home based.** Terms representing the intervention delivery environment (home-based).

Retrieved search results were Imported into Endnote Version x8 and Covidence systematic review software duplicates were removed [20]. Two reviewers independently screened the articles’ titles and abstracts, considering the eligibility criteria in Table 1. The full texts of all potentially eligible articles were screened independently by both reviewers to check eligibility further. Any variance in eligibility assessments between the two reviewers (at either stage of the screening) was discussed to reach an agreement.

### Data extraction and synthesis

Two reviewers extracted data from the studies using a data extraction template. We extracted data on study characteristics, comprising of the lead author, year of publication, country, population, Study design, number of participants), the study aim(s), and intervention characteristics /home-based exercise components. We also extracted data on the definition of adherence used, adherence measures, measurement procedure and any data on measurement reliability, validity, or acceptability and completion /dropout rates for the fidelity measure(s). We conducted a narrative synthesis of the data, including tabulation and narrative summary of the data, structured according to the components of a comprehensive definition of exercise fidelity, which (building on our initial definition above) evolved as the analysis progressed (Table 2).

## Results

### Study inclusion

**Fig. 1:**
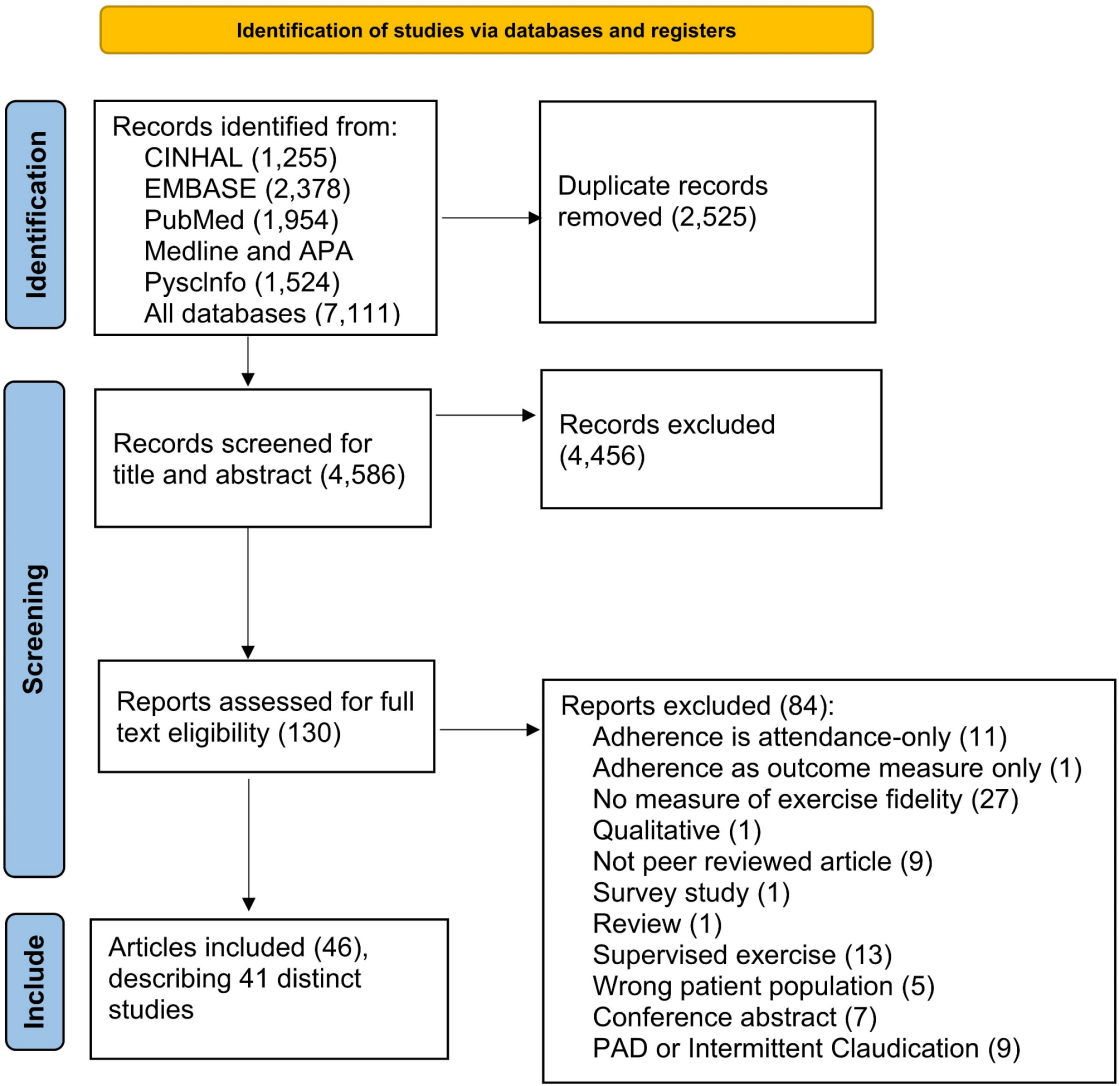
PRISMA flowchart

A PRISMA flowchart is presented in Fig. 1, summarising the study selection process [21]. The search retrieved a total of 4,586 studies after duplicates were removed. After screening titles and abstracts and then full texts, 46 articles describing 41 distinct studies met the inclusion criteria [22–67].

### Characteristics of included studies

The characteristics of the included studies are presented in Table A1 in Additional File 1 and are summarised below:

### Country and population

Most of the studies were conducted in European countries (18), the United States (7), China (6) and Australia (6). The remaining studies were conducted in Israel (2), Portugal (1) and Kenya (1). The study populations included heart failure patients (12), and other cardiovascular populations, including people with coronary artery disease, myocardial infarction and angina.

### Study design /aim and number of participants

The most common study designs were randomised controlled trials (16) and RCT protocols (15) and cohort studies (5) aiming to evaluate the effects of exercise interventions. In these studies, exercise adherence was typically measured as a secondary outcome or as a process measure rather than being the primary objective of the study. Other study designs included RCT pilot and feasibility studies whose aims sometimes included the assessment of exercise adherence. The studies varied widely in sample size (from 12 to 2331).

### Intervention characteristics

The exercise programmes in the included studies varied in duration (from 30 days to 9 months). However, onestudy did not mention the duration of the intervention. The most common intervention setting was home-based (in 31 studies). The remaining ten studies used a hybrid delivery model (a combination of centre-based and home-based cardiac rehabilitation).

### Exercise components

In terms of the exercise components used in the interventions, 16 studies used aerobic exercise only (e.g., cycling or walking), and 17 studies used a combination of aerobic (e.g., walking) and muscle-strengthening /resistance exercise. One study used high-intensity interval training, one study used either high-intensity interval training or moderate-intensity continuous training and one study used stepping exercises. Five studies did not provide any clear information about the exercise components of the interventions.

### Measures used to assess exercise fidelity

This section summarises the measures used in studies to assess the following six components of exercise fidelity considered in this review: Frequency, Intensity, Time, Type /Quality of exercise (accuracy of movement), Safety procedures, and Progression/Regression. The “Time” component was relabeled as “Quantity” to incorporate non-time-based measures of the quantity of exercise undertaken (e.g. steps or distance). A seventh component, ‘Appropriateness of Progression /Regression’, was identified as the analysis progressed: The authors considered that it was important to distinguish between measuring whether (or how often) progression or regression occurred and whether the progression or regression was appropriate (e.g. following a criterion set by the prescribing therapist, such as when your effort score (or heart rate) drops below a certain number, you should progress the exercise to a higher intensity or more difficult type of exercise).

### Frequency of exercise

The majority of studies (32 of the 41) assessed adherence to the prescribed frequency of exercise sessions undertaken by the participants and some studies used multiple measures. The remaining 9 articles either did not measure exercise frequency or provided no clear information about the measures used.

In terms of the measures used, 11 studies used wearable devices to assess exercise frequency. Fourteen studies used exercise or activity diaries, logbooks, or questionnaires (and one study used digital tools to record the questionnaire data). One study used a virtual training app and one used information from phone calls and/interviews. Seven studies assessed session frequency from an electrocardiogram (ECG) data. None of the studies cited evidence on the reliability and validity of measures used for measuring frequency of exercise.

### Intensity of exercise

The majority of studies (38 of the 41) assessed adherence to the prescribed intensity of exercise sessions and some studies used multiple measures. Three studies either did not measure exercise intensity or provided no clear information about the measures used.

Twenty- eight studies used wearable devices, often with a range of measurement functions, including the measurement of heart rate, or steps per minute or (via accelerometry) minutes of activity at selected intensities to measure exercise intensity. Three studies cited evidence for the reliability and validity of the measures used.

Eleven studies used ECG devices, twelve used the Borg scale or another rating of perceived exertion (RPE) scale. One study used software, which converted questionnaire data into metabolic equivalent of task (MET) values. None of these studies cited evidence on the reliability and validity of the measures used.

### Quantity of exercise

#### Duration/time

The majority (24 of the 41) of studies assessed adherence to the intended time-duration of exercise sessions and some studies used multiple measures. The remaining 17 studies did not measure adherence to the intended time-duration and provided no clear information about the measures used.

In terms of the measures used, 15 studies used exercise or activity diaries, logbooks or questionnaires (four studies used digital tools (a web-based data-entry system or an app) to enter data), eight studies used wearable devices. Three studies used ECG. One study used data from a pedometer or a step activity monitor. One study used accelerometer data. One of the above studies was a validation study and found a significant correlation between diary data on exercise duration and exercise capacity (6-minute walk test). The remaining studies did not cite evidence on the reliability and validity of the measures used to assess exercise duration.

### Step count

Ten of the 41 studies measured step counts as an indicator of the quantity of walking exercise. Four studies did not include walking exercises in their programme. The remaining 27 studies did not measure the quantity of walking exercise or provided no clear information about the measures used.

In terms of the type of measures used, nine studies used wearable devices (step activity counter or accelerometer). One study used an *“aerobic stepper”* to count vertical steps. Only one of the above studies provided information about the reliability and validity of the measure used to count steps.

### Distance

Seven studies measured adherence to a prescribed walking distance. Four studies did not include walking exercises. The remaining 30 studies did not assess adherence to the prescribed distance of walking or did not provide clear information about the measures used. No other distance-based exercise prescriptions were used (e.g., swimming, jogging).

In terms of the type of measures used to assess adherence to a prescribed walking distance, seven studies used either a wearable device or a step activity monitor (and step-length data or assumptions about step length) to calculate distance walked. Four studies used either exercise or activity diaries, logbooks, or activity logs. One study used a lap counter, and one used a wearable sensor (one lead ECG) that collects the distance via geospatial location tracking. One study was a validation study that found a significant correlation between pedometer-derived distance walked, and exercise capacity (6-minute walk test). No other studies cited or presented any information about reliability and validity of the measures used for distance.

### Type and quality of exercise

Fifteen studies used diaries /activity logs to record the type of exercise performed. The use of pedometers/counting of steps (in 10 studies) was also considered to implicitly verify that walking had occurred.

None of the 41 studies assessed the quality of exercise performance (i.e., the accuracy of movement in relation to the exercise(s) prescribed). One study used an avatar coach and camera-generated observation to guide the quality of exercise, although no assessment of the exercise quality was reported.

### Safety

Twelve studies measured exercise safety in some way. The remaining studies (29/41) did not assess the safety of exercise(s) undertaken or provided no clear information about the measures used.

In terms of the type of measures used, nine studies assessed the safety of exercise sessions using ECG data (including any abnormal alteration of heart rate or blood pressure, palpation, chest pain, arrhythmia, or dizziness and checking whether exercise was within a prescribed heart rate zone). Two studies used heart rate monitor data to compare heart rate with a specified safety criterion (HR ≤ 80% of HRmax). Two studies used data on blood pressure and heart rate and also recorded signs or symptoms during exercise (self-reported heart distress or dyspnea) and before each exercise session (for safety screening). None of the above studies cited or provided evidence on the reliability and validity of their exercise safety measures.

### Progression/Regression

None of the 41 studies assessed adherence to the intended progression or regression of exercise or provided any clear information about the measures used.

### Appropriateness of progression/regression

None of the 41 studies assessed or reported the appropriateness of progression or regression of exercise sessions (e.g., whether any progressions or regressions made by the participant were appropriate /in accordance with the progression/regression instructions they had been given).

### Definitions of exercise fidelity

Most of the studies (24/41) provided some definition of “adherence”, although there was considerable heterogeneity in the definitions used. Some studies limited their definition to only one or two components of exercise fidelity (e.g., frequency and intensity). The remaining 17 studies either did not define adherence or provided no clear definition.

Following our analysis, we refined our initial definition of exercise fidelity (see Introduction) into a more comprehensive definition that incorporates all the conceptual content of the definitions used in the studies reviewed (see Table 2).

**Table 2:** A comprehensive definition of exercise fidelity

“The degree of adherence to the prescribed exercise programme in terms of the following seven components:”
**1. Frequency**:
The proportion, number, or frequency of prescribed exercise sessions that the participant has attempted.
**2. Intensity**
The proportion or number of minutes spent at the prescribed level of exercise intensity. Intensity can be represented in different terms, including heart rate reserve (HRR), perceived effort (effort scales) and maximum oxygen consumption (VO_2_ max). Intensity for resistance exercise may be expressed in terms of measured resistance or perceived effort (e.g., % of 1-rep max).
**3. Quantity**:
The amount of time that participants spent doing prescribed exercises, or other indicators of exercise quantity (e.g. distance walked, steps, lengths, repetitions, sets). Exercise quantity may be aggregated per session, per week or over other time periods. This should exclude (or be measured separately too) the warm-up and cool-down components of the exercise session.
**4. Type/Quality of exercise (accuracy of movement)**:
Adherence of the participant to the specific types of exercise prescribed (e.g., walking, prescribed strength and balance exercises). For muscle-strengthening or functional exercises, this may also include the accuracy of body movements that the participant attempted to perform in relation to the body movements prescribed.
**5. Safety**:
Exercise safety may include several components. This includes: a) Adherence to the warm-up and cool-down components during all exercise sessions. b) Monitoring for any unwanted signs or symptoms (e.g. arrhythmia, chest pain) before, during, and after each exercise session. c) Exercising above the prescribed intensity limit. d) Movements that may be considered dangerous, or that put participants at risk (e.g., risk of falling).
**6. Progression/Regression**:
The participant’s progression (increasing the aerobic or muscular challenge presented by the prescribed exercise) or regression (decreasing the challenge) of the frequency, intensity, quantity or type of each exercise in line with the prescribed instructions. Progression or regression may be guided by assessment of perceived exertion, intensity, or functional ability [68, 69].
**7. Appropriateness of progression/Regression**:
The extent to which any progression or regression of exercise by the participant was commensurate with instructions for progression/regression provided by the practitioner, or the study protocol, or with general recommendations for exercise progression or regression. For example, ACSM recommends a gradual increase in frequency, intensity, time or type to prevent fatigue, injury or soreness of muscles and any risk at a longer period (17). Regression may be needed if the participant has had a setback in the progression of their fitness or physical functioning (e.g., a bout of illness or break from exercise).

### Feasibility/acceptability of measures of exercise fidelity

**Quantitative measures** (completion rates): Most of the articles (38/41) provided no quantitative information on completion rates for the fidelity measures used. Fifteen of these studies did not provide data as they were RCT protocols. The remaining three studies assessed completion or dropout rates for at least one measure of exercise fidelity (see Table A2 in Additional File 1 for details).

**Qualitative feedback** (comments on feasibility /acceptability or limitations): Twenty-three studies out of 41 provided qualitative feedback about feasibility, acceptability, or limitations related to their measures of exercise fidelity.

The qualitative feedback is summarised in Table A2 in Additional File 1. Briefly, we identified several technical feasibility issues including device battery life, limited internet connectivity and difficulty updating software with PCs or laptops (as opposed to smartphones) and the need to provide support for technical issues with devices and synchronisation. Skin reactions to device materials (e.g., ECG electrodes) and anxiety about interruptions to exercise from device-based safety algorithms were also reported.

Issues with the acceptability of measures included measurement burden (some studies identified the use of heart rate monitors, logbooks, and pedometers as being onerous for some participants). In contrast, other studies reported that using pedometers, wrist-worn devices or smartphones were easy for participants (and that pedometers were relatively low cost). One study reported that presenting a diary as a web application made it accessible for both participants and clinicians.

## Discussion

This review aimed to identify the range, validity, and acceptability of measures for assessing exercise fidelity in unsupervised, home-based cardiovascular rehabilitation. Adherence to exercise frequency was mostly assessed subjectively by self-report (e.g., by exercise diary) and objectively by wearable devices (e.g. heart rate monitors or wearable watches). However, none of the studies reported (or collected) information about the reliability and validity of the measures used. Wearable devices were the most common method for assessing adherence to the intensity of aerobic exercise. However, the intensity of resistance exercise was rarely assessed. Adherence to duration or time of exercise was mainly assessed using exercise diaries, with wearable devices being used in some studies. Step counts and distance were mainly assessed by a pedometer, accelerometer or exercise diary.

Most studies did not assess adherence to exercise safety which might be crucial for patients’ safety during exercise and delivering the interventions in real-world homes based on cardiovascular rehabilitation. Where safety was assessed, this was mainly done (e.g., using a portable ECG device). None of the studies.

assessed adherence to the intended progression or regression of exercise and no studies assessed the quality of exercise (accuracy of movement), or appropriateness of progression or regression.

**Fig. 2:**
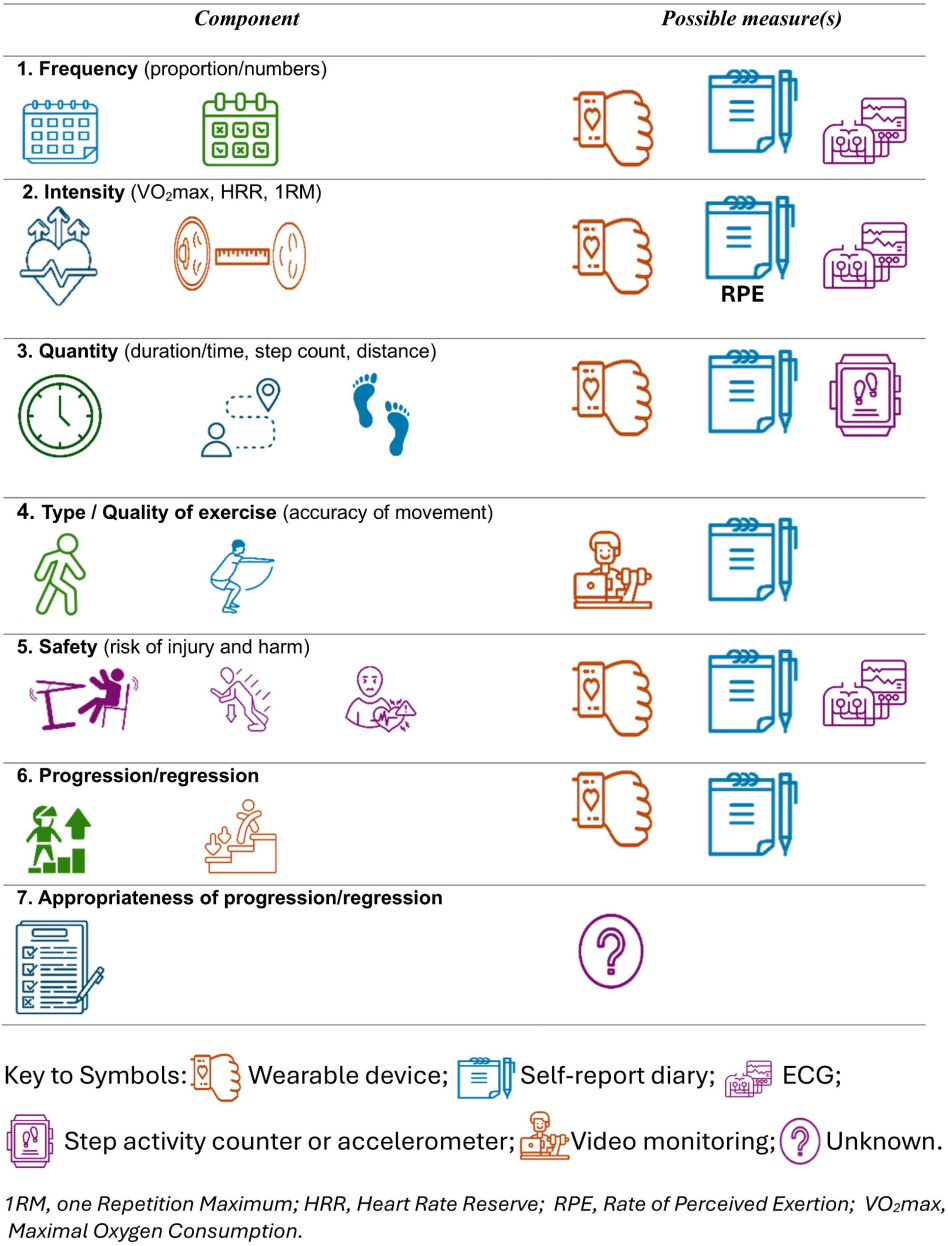
Exercise fidelity components and possible measures

Studies were heterogeneous in the definitions of adherence used. Some studies limited their definition to one or two components of exercise fidelity (often simply defining adherence as the number of exercise sessions undertaken), and some studies did not provide any clear definition of adherence. The variation in definitions of exercise fidelity or adherence prompted us to generate a more comprehensive definition, which synthesises the definitions identified in the review, along with our a priori definition outlined in the Introduction. This comprehensive taxonomy of exercise fidelity components may be useful in guiding future studies and is shown in Table 2 above and Fig. 2 below.

Our finding that most studies used exercise diaries to assess adherence to prescribed exercise frequency is consistent with a previous systematic review of adherence of measures in trials of home-based exercise rehabilitation [14]. However, our review shows that wearable devices such as wrist-worn accelerometers were also used for this purpose. A possible explanation for this is that our scoping review was specifically focused on cardiovascular populations, where wearable devices may be more widely used. Our finding that studies often fail to provide evidence of psychometric validity and reliability for self-assessment questionnaires also agrees with a prior systematic review of self-administered adherence measures for unsupervised home rehabilitation [13].

### Strengths and limitations

Our review is the first to assess how studies of unsupervised/home-based exercise for cardiovascular rehabilitation have assessed exercise fidelity considering all components of a professional exercise prescription, including not only frequency, intensity, time and other measures of quantity type and also quality of exercise performance, progression/regression of exercise, appropriateness of progression/regression and safety of exercise. Our review was systematic, with a search strategy across relevant databases and with multiple coders for the study selection and data extraction processes.

Our review has a number of limitations which should be acknowledged. Our searches were limited to only home-based exercise in cardiovascular rehabilitation and to papers published in the English language only. Therefore, our results result may not generalise fully to other populations and contexts. Further limitations may arise from the quality of reporting in the studies included in our review [70]. 

### Implications for further research or practice

Accurately assessing exercise fidelity is an important issue for future research and practice in home-based interventions that include unsupervised training at home. Greater efforts are needed to assess more comprehensively whether patients did the prescribed exercise(s) as intended, in terms of frequency, intensity, type, quantity, safety and appropriate progression or regression. Otherwise, it is not possible to interpret the findings of home-based exercise intervention trials or other evaluation studies. A failure to find an effect may arise from a lack of efficacy of the prescribed exercise regime, or it might arise from a lack of exercise fidelity [71]. Monitoring of exercise fidelity (in all its aspects) is also a critical issue for clinical practice and guiding of adjustments to the exercise prescribed to individual patients in home-based cardiac rehabilitation (and other home-based exercise) programmes. For example, home-based exercise may be prescribed as part of stroke rehabilitation [72], pre- and post-operative rehabilitation [73, 74], physiotherapy treatments for joint problems [75], self-care programmes for chronic illnesses that may benefit from exercise such as diabetes and cardiovascular conditions [76] as well as in more general public health applications.

Further work is needed to establish robust measures of progression /regression of exercise and appropriateness of progression and regression of exercise. Furthermore, studies should provide more data on the feasibility or acceptability of measures used, which would help inform the choice of exercise fidelity measures for future studies (and future clinical practice). A possible option to assess the quality of exercise would be developing machine learning or sensors to capture the movement of participants during home-based exercise and compare that to reference data on the correct movements for each exercise [77]. We suggest using the comprehensive taxonomy of exercise fidelity components presented in Table 2 to assess exercise fidelity in future studies of home-based cardiovascular rehabilitation programmes. This may also be useful for studies of other home-based exercise programmes, such as those listed above.

## Conclusion

In conclusion, most studies of home-based exercise in cardiac rehabilitation settings have conceptualised exercise fidelity in a very narrow way, often with a focus solely on attendance, with many studies relying on non-validated self-report measures. Future work is needed to develop robust measures to assess the quality, quantity and safety of exercise accuracy of movement, as well as to assess the progression or regression of exercise and the appropriateness of such adjustments. The comprehensive multi-component taxonomy we have presented here provides a potentially useful framework for designing more robust assessments of exercise fidelity and identifying ways to improve fidelity (and thereby effectiveness) in home-based exercise programmes. This may be useful to guide the assessment of exercise fidelity in future research studies and monitoring of exercise fidelity in clinical practice.

## Electronic supplementary material

Below is the link to the electronic supplementary material.

Supplementary Material 1

## Data Availability

The authors confirm that all data generated or analysed during this study is included in this published article.
