# Correction: Methods for assessing exercise fidelity in unsupervised home-based cardiovascular rehabilitation: a scoping review

**DOI:** 10.1186/s13102-025-01235-x

**Published:** 2025-07-01

**Authors:** Mohammad Jarallah, Thomas M Withers, Sheeba Rosewilliam, Afroditi Stathi, Colin J Greaves

**Affiliations:** 1 School of Sport, Exercise and Rehabilitation Sciences, University of Birmingham, Edgbaston, Birmingham, B15 2TT UK; 2 Department of Physical Therapy and Health Rehabilitation, College of Applied Medical Sciences, Majmaah University, Majmaah, 11952 Kingdom of Saudi Arabia

**Correction:** ***BMC Sports Sci Med Rehabil*** **17, 31 (2025)**

10.1186/s13102-025-01069-7

Following the publication of the original article [1], the authors reported that some numbers in the text were mistakenly formatted as reference citations. In the paragraph under the subheading “Country and population”, the bracketed numbers are intended to indicate the number of studies conducted in each country. They are not meant to serve as in-text citations.

Similarly, in the paragraph under the subheading “Study design/aim and number of participants,” the bracketed numbers are intended to represent the number of times a particular study design was used. They are not meant to serve as in-text citations.

The updated section is provided below, with the changes highlighted in bold typeface.

**Incorrect:**

**Country and population**

Most of the studies were conducted in European countries [18], the United States [7], China [6] and Australia [6]. The remaining studies were conducted in Israel [2], Portugal [1] and Kenya [1]. The study populations included heart failure patients [12], and other cardiovascular populations, including people with coronary artery disease, myocardial infarction and angina.

**Study design /aim and number of participants**

The most common study designs were randomised controlled trials [16] and RCT protocols [15] and cohort studies [5] aiming to evaluate the effects of exercise interventions. In these studies, exercise adherence was typically measured as a secondary outcome or as a process measure rather than being the primary objective of the study. Other study designs included RCT pilot and feasibility studies whose aims sometimes included the assessment of exercise adherence. The studies varied widely in sample size (from 12 to 2331).

**Correct:**

**Country and population**

Most of the studies were conducted in European countries **(18)**, the United States **(7)**, China **(6)** and Australia **(6)**. The remaining studies were conducted in Israel **(2)**, Portugal **(1)** and Kenya **(1)**. The study populations included heart failure patients **(12)**, and other cardiovascular populations, including people with coronary artery disease, myocardial infarction and angina.

**Study design /aim and number of participants**

The most common study designs were randomised controlled trials **(16)** and RCT protocols **(15)** and cohort studies **(5)** aiming to evaluate the effects of exercise interventions. In these studies, exercise adherence was typically measured as a secondary outcome or as a process measure rather than being the primary objective of the study. Other study designs included RCT pilot and feasibility studies whose aims sometimes included the assessment of exercise adherence. The studies varied widely in sample size (from 12 to 2331).

The original article [1] has been corrected.
