# Supplementary material for: Methods for assessing exercise fidelity in unsupervised home-based cardiovascular rehabilitation: a scoping review
Source: BMC Sports Sci Med Rehabil. 2025 Feb 28;17:31. doi: 10.1186/s13102-025-01069-7 (PMC11871813; doi:10.1186/s13102-025-01069-7)
Supplement: Supplementary file 1 — Supplementary Material 1 [file 13102_2025_1069_MOESM1_ESM.docx]

# ADDITIONAL FILE 1

# Methods for assessing exercise fidelity in unsupervised home-based cardiovascular rehabilitation: A scoping review.

Jarallah JM, Withers TM, Rosewilliam S, Stathi A, Greaves CJ

1. **Search strategy and (EMBASE) results**

Database: Embase <1974 to 2021 July 26>

| # | Results  Query from 27  Jul 2021 | |
| --- | --- | --- |
| 1 | Exercis*.mp. [mp=title, abstract, heading word, drug trade name, original title, device manufacturer, drug manufacturer, device trade name, keyword, floating subheading word, candidate term word] | 564,123 |
| 2 | limit 1 to (human and english language) | 439,621 |
| 3 | "physical activity".mp. [mp=title, abstract, heading word, drug trade name, original title, device manufacturer, drug manufacturer, device trade name, keyword, floating subheading word, candidate term word] | 234,379 |
| 4 | limit 3 to (human and english language) | 206,921 |
| 5 | walking.mp. [mp=title, abstract, heading word, drug trade name, original title, device manufacturer, drug manufacturer, device trade name, keyword, floating subheading word, candidate term word] | 146,810 |
| 6 | limit 5 to (human and english language) | 120,922 |
| 7 | Resistance training.mp. [mp=title, abstract, heading word, drug trade name, original title, device manufacturer, drug manufacturer, device trade name, keyword, floating subheading word, candidate term word] | 24,527 |
| 8 | limit 7 to (human and english language) | 22,407 |
| 9 | Strength training.mp. [mp=title, abstract, heading word, drug trade name, original title, device manufacturer, drug manufacturer, device trade name, keyword, floating subheading word, candidate term word] | 7,644 |
| 10 | limit 9 to (human and english language) | 6,848 |
| 11 | Muscle strengthening.mp. [mp=title, abstract, heading word, drug trade name, original title, device manufacturer, drug manufacturer, device trade name, keyword, floating subheading word, candidate term word] | 1,798 |
| 12 | limit 11 to (human and english language) | 1,606 |
| 13 | Adher*.mp. [mp=title, abstract, heading word, drug trade name, original title, device manufacturer, drug manufacturer, device trade name, keyword, floating subheading word, candidate term word] | 328,252 |
| 14 | limit 13 to (human and english language) | 237,536 |
| 15 | Complian*.mp. [mp=title, abstract, heading word, drug trade name, original title, device manufacturer, drug manufacturer, device trade name, keyword, floating subheading word, candidate term word] | 373,850 |
| 16 | limit 15 to (human and english language) | 299,239 |
| 17 | Fidelity.mp. [mp=title, abstract, heading word, drug trade name, original title, device manufacturer, drug manufacturer, device trade | 35,809 |

|  | name, keyword, floating subheading word, candidate term word] |  |
| --- | --- | --- |
| 18 | limit 17 to (human and english language) | 20,565 |
| 19 | (Home adj2 Deliver*).mp. [mp=title, abstract, heading word, drug trade name, original title, device manufacturer, drug manufacturer, device trade name, keyword, floating subheading word, candidate term word] | 6,625 |
| 20 | limit 19 to (human and english language) | 5,854 |
| 21 | Home-based.mp. [mp=title, abstract, heading word, drug trade name, original title, device manufacturer, drug manufacturer, device trade name, keyword, floating subheading word, candidate term word] | 16,582 |
| 22 | limit 21 to (human and english language) | 15,431 |
| 23 | Home based.mp. [mp=title, abstract, heading word, drug trade name, original title, device manufacturer, drug manufacturer, device trade name, keyword, floating subheading word, candidate term word] | 16,582 |
| 24 | limit 23 to (human and english language) | 15,431 |
| 25 | Non-supervised.mp. [mp=title, abstract, heading word, drug trade name, original title, device manufacturer, drug manufacturer, device trade name, keyword, floating subheading word, candidate term word] | 374 |
| 26 | limit 25 to (human and english language) | 262 |
| 27 | Non supervised.mp. [mp=title, abstract, heading word, drug trade name, original title, device manufacturer, drug manufacturer, device trade name, keyword, floating subheading word, candidate term word] | 374 |
| 28 | limit 27 to (human and english language) | 262 |
| 29 | Self deliver*.mp. [mp=title, abstract, heading word, drug trade name, original title, device manufacturer, drug manufacturer, device trade name, keyword, floating subheading word, candidate term word] | 331 |
| 30 | limit 29 to (human and english language) | 193 |
| 31 | Self-deliver*.mp. [mp=title, abstract, heading word, drug trade name, original title, device manufacturer, drug manufacturer, device trade name, keyword, floating subheading word, candidate term word] | 331 |
| 32 | limit 31 to (human and english language) | 193 |
| 33 | exp exercise/ | 367,152 |
| 34 | limit 33 to (human and english language) | 298,470 |
| 35 | exp muscle stretching/ | 5,525 |
| 36 | limit 35 to (human and english language) | 3,123 |
| 37 | exp resistance training/ | 21,218 |
| 38 | limit 37 to (human and english language) | 19,466 |
| 39 | exp physical fitness/ | 38,439 |
| 40 | limit 39 to (human and english language) | 29,988 |
| 41 | exp patient Compliance/ | 168,089 |
| 42 | limit 41 to (human and english language) | 149,495 |
| 43 | 2 or 4 or 6 or 8 or 10 or 12 or 34 or 36 or 38 or 40 | 681,508 |
| 44 | 14 or 16 or 18 or 42 | 484,046 |
| 45 | 20 or 22 or 24 or 26 or 28 or 30 or 32 | 21,358 |
| 46 | 43 and 44 and 45 | 1,426 |

Exercis*.mp. [mp=title, abstract, heading word, drug trade name, original title, device manufacturer, drug manufacturer, device trade name, keyword, floating subheading word, candidate term word]

limit 1 to (human and english language)

"physical activity".mp. [mp=title, abstract, heading word, drug trade name, original title, device manufacturer, drug manufacturer, device trade name, keyword, floating subheading word, candidate term word]

limit 3 to (human and english language)

walking.mp. [mp=title, abstract, heading word, drug trade name, original title, device manufacturer, drug manufacturer, device trade name, keyword, floating subheading word, candidate term word]

limit 5 to (human and english language)

Resistance training.mp. [mp=title, abstract, heading word, drug trade name, original title, device manufacturer, drug manufacturer, device trade name, keyword, floating subheading word, candidate term word]

limit 7 to (human and english language)

Strength training.mp. [mp=title, abstract, heading word, drug trade name, original title, device manufacturer, drug manufacturer, device trade name, keyword, floating subheading word, candidate term word]

limit 9 to (human and english language)

Muscle strengthening.mp. [mp=title, abstract, heading word, drug trade name, original title, device manufacturer, drug manufacturer, device trade name, keyword, floating subheading word, candidate term word]

limit 11 to (human and english language)

Adher*.mp. [mp=title, abstract, heading word, drug trade name, original title, device manufacturer, drug manufacturer, device trade name, keyword, floating subheading word, candidate term word]

limit 13 to (human and english language)

Complian*.mp. [mp=title, abstract, heading word, drug trade name, original title, device manufacturer, drug manufacturer, device trade name, keyword, floating subheading word, candidate term word]

limit 15 to (human and english language)

Fidelity.mp. [mp=title, abstract, heading word, drug trade name, original title, device manufacturer, drug manufacturer, device trade name, keyword, floating subheading word, candidate term word]

limit 17 to (human and english language)

(Home adj2 Deliver*).mp. [mp=title, abstract, heading word, drug trade name, original title, device manufacturer, drug manufacturer, device trade name, keyword, floating subheading word, candidate term word]

limit 19 to (human and english language)

Home-based.mp. [mp=title, abstract, heading word, drug trade name, original title, device manufacturer, drug manufacturer, device trade name, keyword, floating subheading word, candidate term word]

limit 21 to (human and english language)

Home based.mp. [mp=title, abstract, heading word, drug trade name, original title, device manufacturer, drug manufacturer, device trade name, keyword, floating subheading word, candidate term word]

limit 23 to (human and english language)

Non-supervised.mp. [mp=title, abstract, heading word, drug trade name, original title, device manufacturer, drug manufacturer, device trade name, keyword, floating subheading word, candidate term word]

limit 25 to (human and english language)

Non supervised.mp. [mp=title, abstract, heading word, drug trade name, original title, device manufacturer, drug manufacturer, device trade name, keyword, floating subheading word, candidate term word]

limit 27 to (human and english language)

Self deliver*.mp. [mp=title, abstract, heading word, drug trade name, original title, device manufacturer, drug manufacturer, device trade name, keyword, floating subheading word, candidate term word]

limit 29 to (human and english language)

Self-deliver*.mp. [mp=title, abstract, heading word, drug trade name, original title, device manufacturer, drug manufacturer, device trade name, keyword, floating subheading word, candidate term word]

limit 31 to (human and english language) exp exercise/

limit 33 to (human and english language) exp muscle stretching/

limit 35 to (human and english language) exp resistance training/

limit 37 to (human and english language) exp physical fitness/

limit 39 to (human and english language) exp patient Compliance/

limit 41 to (human and english language)

2 or 4 or 6 or 8 or 10 or 12 or 34 or 36 or 38 or 40

14 or 16 or 18 or 42

20 or 22 or 24 or 26 or 28 or 30 or 32

43 and 44 and 45

**Table A1: Characteristics of included studies.**

| **Lead author (year)** | **Country and Population** | **Study design, number of participants, study aim.** | **Intervention characteristics** | **Home-based exercise components** |
| --- | --- | --- | --- | --- |
| Aamot et al. (2016)  (1) | **Country:** Norway  **Population:**  Adults eligible for cardiac rehabilitation | RCT follow-up study  N=90, 76 with usable data  **Aim:** To assess long-term (12 months) adherence to exercise following completion of a cardiac rehabilitation (CR) programme. | Unsupervised home-based exercise following an initial 12-week high-intensity exercise (HIIT) programme where patients were trained to work at 85-90% of HR max in bouts of 4 mins. | Home-based HIIT twice weekly (as specified in the initial intervention), or if preferred, alternative non-HIIT exercise to meet physical activity recommendations (30 mins /day of MVPA). |
| Batalik et al. (2018)  Batalik et al. (2020)  (2, 3) | **Country**: Czech Republic  **Population:**  CAD, CVD (MI, angina pectoris) | RCT Protocol and RCT  N= 56,28 in intervention arm  **Aims:**  1-To evaluate the effectiveness of a hybrid (part centre-, part home-based) exercise training programme on quality of life and physical fitness.  2-To explore the efficacy of using wearable heart rate monitors for improving exercise adherence compared to outpatient CR. | Centre-based exercise for 2 sessions, then home-based exercise for 10 weeks. | The exercise components were:  a) 10 mins warm-up.  b) 60 mins cycling or walking with approximately 70-80% of heart rate reserve.  c) 10 mins cool down.  d) Three sessions per week for 60 mins for every session. |
| Bernocchi et al. (2016)(4) | **Country:** Italy  **Population:**  COPD and CHF | RCT protocol  N= 144, 77 in intervention arm  **Aim:** To explore the effectiveness and feasibility of home-based telehealth rehabilitation compared to usual care. | Home-based telehealth programme for 4 months delivered after initial inpatient assessment and education (which controls also received). | The exercise components were:  1- “light training” including:  a) Free weight mini ergometer: 15-25 mins three days per week.  b) Walking: Two sessions per week  c) Calisthenic exercise: Three sets weekly.  2- *“hard training”*  a) 0 to 60 watts on mini ergometer: 30-45 minutes 3-7 days per week.  b) Walking: 3-7 sessions per week.  c) Muscle strengthening with 0.5-kilo weights 3-7 sessions per week.  The target intensity was to achieve a moderate to high level of dyspnoea or muscle fatigue. Diarised assessments guided progression or regression of intensity via weekly phone calls with a physiotherapist. |
| Brouwers et al. (2017)  (5) | **Country:** Netherlands  **Population:** CAD | RCT protocol  N= 300, 150 in intervention arm  **Aim:** To investigate whether cardiac telerehabilitation and multiple behavioural change strategies increase long-term physical activity levels compared with centre-based CR. | Six supervised outpatient training sessions followed by Web application supported home cardiac rehabilitation, with weekly video consultation for 3 months, followed by 9 months of on-demand coaching. | Individualised exercise prescription including:  1-Functional and resistance exercise.  2-Aerobic exercises (high-intensity or continuous training).  3-Duration ranged from 20- to 60 minutes.  4- Intensity: 80-90% of HRR for high-intensity at 50-80% of HRR for continuous training. |
| Cai et al. (2022)  (6) | Country: China  Population: AF | RCT  N=97, 49 in intervention arm  **Aim:** To assess short-term effectiveness of cardiac tele-rehab on exercise capacity compared to conventional CR. | App guided home-based exercise for 12 weeks. | The exercise components were:  a) 10 mins warm-up and cool-down.  b) 45 mins jogging, fast walking, etc., at HR target.  c) 12 weeks with 150 min of exercise per week. Number of sessions is not specified. |
| Claes et al. (2017)  (7) | **Countries:** Ireland and Belgium  **Population:** CVD | Single-blind two-group pilot RCT  N=120, 60 in intervention group **Aim:** To assess intervention effects on objectively measured active energy expenditure at six months from baseline. | In addition to 2-3 weekly CR exercise sessions: A weekly familiarisation session for each of the last four weeks of CR, then (indefinitely), a technology-driven self-care CR system. | a) Exercise module and an outdoor physical activity module.  b) Exercise prescriptions were individualised and included exercise classes (aerobic, resistance and relaxation) and exercise games (including feedback on accuracy of movement via movement capture). |
| Ding et al. (2021)  (8) | **Country:** United States  **Population:** MI | Feasibility study  N=18  **Aim:** To assess the feasibility of MI-PACE a home-based cardiac telerehabilitation programme. | Home-based telerehabilitation for 12 weeks with weekly nurse-led sessions to review progress and set exercise goals. | a) Individually tailored number of walking sessions and moderate to vigorous sessions.  b) Completion of a goal prompted an increase in frequency or duration of sessions.  c) Intensity was based on the difference between resting heart rate and peak heart rate along with exercise stress test results. |
| Dor-Haim et al. (2019)  (9) | **Country:** Israel  **Population** Ischemic heart disease | RCT protocol  N= 60 in intervention arm  **Aim:** To assess the effect on long-term adherence to PA in CR of adding digital monitoring to usual CR. | Centre-based CR for 6 weeks, followed by 6 weeks of home-based telemonitoring with monitoring via a wearable device CR for 12 weeks. | Exercise 5 days a week (for a total of 150 minutes) at 60% of target heart rate. |
| Dougherty et al. (2016)  (10) | **Country:** USA  **Population:** ICD patients with an implantable cardioverter-defibrillator | RCT  N=160, 84 in intervention arm  **Aim:** To explore the effectiveness of the intervention on adherence to home-based aerobic exercises. | Eight weeks of home-based aerobic conditioning followed by 16 weeks of aerobic maintenance. | Eight weeks of aerobic conditioning with walking 60 mins per day for five days per week or 300 mins per week. This is followed by 16 weeks with a minimum of 150 mins per week and at least 30 mins per session.  Target heart rate reserve for each session was 60-65% in weeks 1-2, 70-75% in weeks 3-4 and 80-85% in weeks 5-24. |
| Du et al. (2011)  (11) | **Country:** Australia  **Population:** CHF | RCT protocol  N= 166, 83 in intervention arm  **Aim:** To evaluate the effect of the home heart walk programme on exercise self-efficacy and self-management. | A home-based walking programme for 6 months. | Walking programme for at least one session per week. |
| Evangelista et al. (2005)  (12) | **Country:** USA  **Population:** HF | Observational study  N= 38  **Aim:** To assess the validity of pedometers as a measure of exercise adherence. | Supervised home-based walking programme for 12 months. | 1-Tailored 45-minute walking at 60% of HR max (frequency not stated).  2-After six weeks, a light resistance component was added. |
| Giallauria et al. (2006)  (13) | **Country**: Italy  **Population:** AMI | Non-randomised control trial  N= 45, 15 in intervention arm  **Aim:** To assess the potential efficacy of telecardiology for improving adherence to exercise prescription. | Eight-week home-based cardiac rehabilitation programme with telecardiology monitoring. | 1-Cycling three times per week for 30 mins at heart rate target around 75% of peak heart rate).  2-Exercise prescription was individually tailored for each participant. |
| Higgins et al. (2001)  (14) | **Country**: Australia  **Population** PCI | RCT  N=99, 50 in intervention arm  **Aim:** To evaluate whether a home-based cardiac rehabilitation programme reduced CV risk factors and speeded up return to work. | A home-based cardiac rehabilitation programme with three home visits. | 1) A walking programme at moderate intensity with gradual progression of duration and frequency of exercise during the intervention. |
| Isakadze et al. (2024)  (15) | Country: US  Population: MI, CABG, TAVR, or valvular heart surgery | RCT protocol  N= 200, 100 for intervention arm  Aim: To evaluate the efficacy of improving functional capacity and safety of Corrie HBCR. | A hybrid CR programme for 12 weeks. | Details of the exercise prescription are not reported. |
| Karjalainen et al. (2012)  (16) | **Country:** Finland  **Population:** CAD with or without type 2 diabetes | RCT  N= 83 (44 CAD, 39 CAD and type 2 diabetes), 83 in intervention arm  **Aim:** To assess whether individually tailored home-based exercise prescription increases long-term regular physical activity. | Six-month home-based exercise programme. | A mixture of endurance training (30 mins) and strength training (30 mins).  ***First three months:***  Three endurance sessions at 50-60% of HRR and one strength exercise session.  ***After three months****:*  Endurance exercise. 2 weekly exercise sessions at 50-60% of HRR. Plus 2 weekly exercise sessions at 60-70% of HRR. Strength exercise for one session per week. |
| Korzeniowska-Kubacka et al. (2015)  (17) | **Country:** Poland  **Population:** MI | Longitudinal cohort study comparing outcomes between men and women.  N=87.  **Aim:** To compare the benefits of hybrid cardiac rehabilitation between men and women. | A hybrid eight-week cardiac rehabilitation programme for 24 sessions with interval training.  1-10 sessions at outpatient.  2-14 sessions at home. | 1) Home-based walking interval training: 10 mins walking followed by 2 mins rest repeated three times and relaxation for 10 mins.  2) Intensity: Between 60-80% of the HR zone. |
| Li et al. (2019)  (18) | **Country:** China  **Population:** CHF | RCT protocol.  N= 120 ,60 per group  **Aim:** To evaluate the effectiveness and safety of home-based exercise rehabilitation using ECG monitoring. | Four weeks of supervised exercise, followed by 8 weeks of home-based, remote ECG-supervised walking. | Eight weeks of home-based walking exercise,40 mins, five times /week at 60-70% heart rate reserve. |
| Li et al. (2015)  (19) | **Country:** China  **Population:** CVD | RCT  N=77 ,37 in intervention arm  **Aim** To assess the benefits of home-based versus centre-based exercise rehabilitation. | A 12-week home-based exercise rehabilitation programme with nurse follow-up. | 1- Functional exercise for a minimum of 5 days /week for approximately 10 mins. Targeting Borg scale 9 -11 and heart rate 10-20 BPM above resting.  2. 20-30 min walking, at least 5 days /week with a 5-min warm-up and cool-down. |
| Liu et al. (2022)  (20) | Country: China  Population: CHD | RCT  N=98  Aim: To assess the effectiveness of adding home-based Tai Chi to centre-based Tai Chi on functional health. | Six-weeks of group Tai Chi followed by six weeks of home-based Tai Chi. | The exercise comprised the following:  1)Tai Chi exercise for 60 mins for 4 times per week at an intensity of12-13 on the Borg Scale.  2)The participants were provided with Tai Chi booklets and Video CDs. |
| Lloyd et al. (2019)  (21) | **Country:** USA  **Population:** HF | Feasibility study, N=12  **Aim:** To assess the feasibility of a web-based assistant targeting medication, weight monitoring and aerobic activity. | A web-based assistant to support medication adherence, weight monitoring and aerobic activity. Home exercise using a ‘stepper’ for 30 days. | Using the stepper daily. |
| Nabutovsky et al. (2024)  (22) | Country: Israel  Population: MI | RCT  N=69, 45 in intervention arm  Aim: To assess the effectiveness of home-based CR on exercise capacity in patients who turned down Centre-based CR. | A home-based Cardiac rehabilitation programme for 6 months. | 1) Two sessions/week of resistance exercise.  2) Advice to attain 8000 steps per day. |
| Ngeno et al. (2022)  (23) | Country: Kenya  Population: HF | Prospective feasibility study  N=100, 31 for the HBCR arm  Aim: To evaluate the feasibility (by assessing adherence) of delivering CBCR and HBCR in West Kenya. | A home-based cardiac rehabilitation programme for 12 weeks. | 1) Participants were advised to do daily brisk walking.  2) Target exercise was increased by 20 minutes every 4 weeks up to 60 minutes at week 12. |
| Nielsen et al. (2019)  (24) | **Country:** USA  **Population:** HFrEF | RCT  N=20, 10 in intervention arm  **Aim:** To evaluate the feasibility of patient selected exercise adherence strategies on exercise adherence and physiological outcomes. | A 12-week home-based exercise adherence intervention. The intervention arm used strategies to improve motivation to exercise, such as exercise logs, educational handouts, nurse phone calls, and letters. | 30 mins plus warm-up and cool-down, 5 times per week, to achieve a rating of perceived exertion (RPE) scale score between 12-13 (exercise type not specified). |
| O’Connor et al. (2009) Jones et al. (2014)  (25, 26) | **Country:** USA  **Population:** Chronic HF | RCT  N=2331, 1159 in intervention arm  **Aims:** 1-To assess whether regular structured exercise reduces all-cause mortality and hospitalisation.  2-To explore the safety and influence of aerobic exercise in a sub-group of 90 cancer patients with stable HF. | Six weeks of centres based, then a combination of centre-based and home (weeks 7-12) and then home-based only. | Cycling or walking:  a) Week 7-12: Twice /week at home for 30-35 mins at 70% heart rate reserve.  b) Week 13 to end: Five times per week for 40 mins at 60-70% heart rate reserve. |
| Oka et al. (2000)  Oka et al. (2005)  (27, 28) | **Country:** USA  **Population:** HF | RCT  N= 40,20 in intervention arm  **Aims:**  1- To evaluate the effectiveness of home-based walking and resistance exercise on 3-month fitness.  2- To assess the effect of treadmill training on “positive mystery” and engagement in exercise training to improve self-efficacy in patients with heart failure. | A home-based 3 months walking and resistance exercise programme. | a) Three days a week walking at intensity that increased to 70% of heart rate peak by the 2^nd^ -3^rd^ week for a duration of 40 to 60 mins.  b) Two days a week of total body resistance exercise increasing to approximately 75% one rep max with a duration of 30-40 min. |
| Pater et al. (2000)  (29) | **Country:** Norway  **Population**: MI, ACS, PTCA, and Coronary artery bypass grafting | RCT protocol  N=500 estimated,250 per group  **Aim:** To assess the long-term effectiveness of a comprehensive cardiac rehabilitation programme. | Eight weeks of supervised physical activity, followed by eight weeks of structured home-based physical activity. | Exercise prescription was individualised for participants in the home-based stage of the programme. Three sessions per week at 60-70% HRmax, 20 mins. The study did not specify the type of exercise or physical training. |
| Piotrowicz et al. (2010)  Piotrowicz et al. (2014)  Piotrowicz et al. (2015)  (30-32) | **Country:** Poland  **Population:** HF | RCT  N=365  **Aim:** To assess the effectiveness of a home-based telemonitored cardiac rehabilitation programme. | Eight-week home-based walking telemonitored programme.  For Piotrowicz et al. (2014)  Four weeks with 2 phases:  1) The first phase is for three days at the centre.  2) The second phase is for four weeks at home. | Light resistance and breathing exercises for warm-up, a continuous walking programme on floor level, aerobic exercises (cycloergometer,“Nordic walking” or ambulation) for 30 mins per session. Aiming to reach 11 on a RPE scale and 40-70% of HR. Ten minutes per session and progressed to 30 mins. Five-minute cool-down using relaxation exercise. Nordic walking initiation based on the baseline of CPET. |
| Rawstorn et al. (2020)  (33) | **Country:** Australia  **Population:** CHD (Coronary revascularisation, MI, angina) | RCT protocol  N= 220 estimated,110 per group  **Aim:** To assess the effectiveness of a smartphone-facilitated cardiac rehabilitation application | A 24-week (12-week intensive and 12 maintenance) home-based exercise programme. | Details of the exercise prescription are not reported |
| Schmidt et al. (2024)  (34) | Country: Portugal  Population: HF | RCT protocol  N=120 initially randomised at 1:1 then 1:2  Aim: To assess the economic and clinical effectiveness of HBCR on exercise capacity compared to CBCR. | Home-based Cardiac rehabilitation for 12 weeks following an initial 4-5 centre-based sessions. | The exercise components were:  a) 5-10 mins warm-up using stretching and calisthenic exercise.  b) 25 min resistance exercise.  c) 30 min Moderate to vigorous aerobic training.  d) 5 mins cool down. In addition, patients were encouraged to walk at home for 30 at least once a week. |
| Shen et al. (2024)  (35) | Country: China  Population: TAVR | RCT protocol  N=90  Aim: To assess the effectiveness of mobile guided exercise-based cardiac rehabilitation on functional capacity compared with usual care. | Home-based Cardiac rehabilitation for 12 weeks. | Individualised exercise prescription aerobic exercise 3-5 days a week:  a) 5-10 mins warm-up.  b) 30 mins of aerobic exercises.  c) 5-10 min cool-down.  d)) Exercise intensity 11-14 on a RPE scale.  e) Resistance exercise: 1-2 days a week with 2-4 sets of 10-15 repetitions per day. |
| Skobel et al. (2017)  (36) | **Countries:** Spain, the UK, and Germany  **Population:** CAD | RCT  N= 118, 55 in intervention arm  **Aim:** To assess the effectiveness of a smartphone-guided training system. | A six-month smartphone-guided home-based exercise programme. | 1- Week 1-3: Endurance exercise twice a week.  2- Week 4-7 Addition of resistance exercise twice a week.  3- Week 8-9 Increase endurance and resistance to three times a week.  4- Thereafter, maintenance endurance exercise 3 times a week to daily and resistance 3 times a week. |
| Smart et al. (2005)  (37) | **Country:** Australia  **Population:** HF | Longitudinal cohort study  N=30  **Aim:** To assess the feasibility of emails, calls, exercise diaries and heart rate monitoring as motivational tools. | Sixteen weeks of centre-based cycle-based training followed by home exercise and telemonitoring. | Week 17 onwards:  1) 15-minute step exercise once a week, 30 mins of 6-minute walks once weekly and a 60-minute walk once a week.  2)Aerobic exercise included the following:  a) *“long walk “,* duration:60 mins, frequency: 1 per week, RPE:3 and to progress by increasing the walking speed.  b*)”6-min walks”,*3 to 5 x 6 mins,1 per week. RPE was 4 in 6 mins and progressed by adding 1 rep per two months.  C)*” Stair climb”* for 15X 30 s.1 session per week. RPE=5, and to progress by decreasing break time and adding 1 set per week. |
| Suchy et al. (2014)  (38) | **Country:** Europe  **Population:** HF | RCT protocol  N= 180, 60 moderate-intensity continuous training,60 High-intensity interval training.  **Aim:** To define the optimal dose of exercise in HF with preserved left ventricular ejection fraction. | Three months supervised, followed by 9 months of home-based exercise training in two groups: high-intensity interval training and moderate-intensity continuous training. A monthly session at the centre for feedback and monitoring. | **Continuous moderate training (MCT)**  Cycle ergometer: 40 mins,5 sessions weekly,60-70% of HRmax, 50-60% of Vo2 max.  **High-intensity interval training HIIT**  A warm-up for 10 mins at 50-60% of Vo2 max,60-70% of HR max, 11-13 score at RPE scale. A cycling exercise for 4 mins at 90-95% of HRmax, 85-90% of Vo2 max, and 15 to 17 score on RPE scale. Interval stop time was 3 mins and cycling intensity was 50-70% of HRmax. A cool down for 3 mins. The duration of the session was 38 mins. |
| Szalewska et al. (2015a)  (39) | **Country:** Poland  **Population:** Cardiovascular disease | Retrospective cohort study  N= 152  **Aim:** To assess whether professional status influences telerehabilitation. | A hybrid consisted of:  Eight to ten days of centre-based rehabilitation followed by 10-to-12-days home-based. | Exercise prescription was individualised per patient, and intensity was 60-80 % of HR, including aerobic exercise for 30 mins using a cycle ergometer, Nordic walking, and normal walking and 5-10 mins warm-up and cool-down. |
| Szalewska et al. (2015b)  (40) | **Country:** Poland  **Population:** CAD with or without diabetic mellitus | Retrospective cohort study  N= 125  **Aim:** To compare outcomes between hybrid cardiac rehabilitation patients with and without diabetes. | 8-10 days centre based, then 11-12 days home-based rehab. | A warm-up and cool-down between 5 to 10 mins at low intensity. Thirty mins of aerobic exercise (Nordic walking, walking or cycle ergometer and flexibility exercise, light resistance and systemic exercise),60-80% of heart rate reserve and aiming between 12-13 on RPE scale. |
| Torri et al. (2018)  (41) | **Country:** Italy  **Population:** CVD (post MI, or percutaneous or surgical coronary vascularisation, or heart failure) | Quasi-experimental study  N=53,26 in intervention arm  **Aim:** To assess the effectiveness and feasibility of web-monitored exercise on physical activity and functional capacity. | Following an inpatient CR programme, patients were offered a home-based exercise programme for 6 months, with web-based information, self-reporting of exercise and intermittent phone support from a physiotherapist (frequency not specified). | a) Aerobic training at light to moderate intensity at RPE scale between 30 to 40 mins, with a minimum frequency of 3 days per week.  b) Resistance exercise with stretch bands or dumbbells for 20 mins while working at 70 to 80% of one repetition maximum (I RM). |
| Varnfield et al. (2014)  (42) | **Country:** Australia  **Population:** post-MI | RCT  N=120, in intervention arm, 60 controls  **Aim:** To assess the effectiveness of using a Care assessment platform (CAP) in a home-based CR programme compared to traditional centre-based CR. | 6 weeks of CAP-CR at home with weekly mentor support, followed by 6 months of self-management. Supported in both phases by smartphone monitoring of health and educational  material. | a) Moderate exercise intensity at 11-13 score on RPE scale for a minimum of 30 mins per day on most days of the week, with walking as the main exercise mode.  b) Relaxation (no details reported). |
| Vonk et al. (2021)  (43) | Country: Netherlands  Population: CAD | RCT protocol  N= 132 for four arms  Aim: To assess the effect of adding HBCR to CBCR on physical activity. | CBCR followed by HBCR in three variants:  1) Six weeks combined CBCR and HBCR.  2) Six weeks CBCR followed by HBCR for 12 weeks.  3) Six weeks combined CBCR and HBCR followed by 12 weeks of HBCR. | a) Daily physical activity using app.  b) Aerobic exercise started at 5-10 min gradually increasing to >30 minutes per day.  c) Resistance exercise 2 times per week, 1-3 sets of 10-15. repetitions or timebound (20 – 120 s). |
| Walters et al. (2010)  (44) | **Country:** Australia  **Population:**  Patients who have had a myocardial infarction (heart attack) | RCT Protocol  N= 100 for intervention arm  **Aim:** To assess the effectiveness of home-based CR when enhanced by novel Information and Communication Technologies. | A six-week home-based CR programme, enhanced with mobile phone app and text-messaging support. | A walking programme at low to moderate intensity for a minimum of 30 mins per session. |
| Webb-Peploe et al. (2000)  (45) | **Country**: UK  **Population:** Stable heart failure | Crossover RCT  N= 24 for intervention arm  **Aim:** To assess the effectiveness of a home-based aerobic exercise programme on patients with stable heart failure. | A home-based exercise programme for 8 weeks (and 8 weeks of rest in the crossover condition). | a) Bicycle ergometer at 50 Revolutions per minute at 70-80% of HR max for a minimum of 5 days per week and 20 mins /day.  b) Callisthenic exercise for a minimum of 5 days per week and 9 mins /day, following the XBX programme which has four progressively difficult exercise charts. Participants progressed the difficulty of exercises once they could complete a level within the 9-minute time limit. |
| Xu et al. (2020)  (46) | **Country:** China  **Population:** CHD | Crossover RCT protocol  N= 70 for randomisation,70 for intervention arm  **Aim:** To assess the effectiveness of the *“Intelligent Exercise Rehabilitation Management System (IERMS)*” on exercise adherence in patients with CHD. | A 6-week home-based cardiac rehabilitation telemonitoring system delivered following a standard outpatient CR programme. Smart insoles monitored the intensity and type of exercise and provided AI-driven feedback to patients and medical staff. | a) Resistance exercise (no details reported).  b) Endurance exercise (no details reported). |

| **CAD:** Coronary artery disease, **MI:** Myocardial infarction, **CVD:**Cardiovascular disease, **COPD:** Chronic obstructive pulmonary disease, **CHF:** Chronic heart failure, **HF:** Heart failure ,**IHD:** Ischaemic heart disease ,**ICD:** Implantable cardioverter defibrillator, **PAD**: Peripheral arterial disease, **AMI:** Acute myocardial infarction, **PCI:** Percutaneous coronary intervention, **HFrEF:** Heart failure with a reduced ejection fraction, **CABAG**: Coronary Artery Bypass Graft, **PTCA:** Percutaneous transluminal coronary angioplasty, **ACS:** Acute coronary syndrome, ,**CHD:** Coronary heart disease**, EF**: Ejection fraction, **LVEF:** Left Ventricular ejection fraction, **HFpEF:** Heart failure with preserved ejection fraction, **NSTEMI:** Non-ST segment elevation myocardial infarction, **STEMI**: ST-Segment Elevation Myocardial Infarction, **RCT:** Randomised controlled trial, **CR:** Cardiac rehabilitation , **HIIT**; High-intensity interval training, **HRR**: Heart rate reserve ,**RPE:** Rated Perceived Exertion, **HR**: Heart rate ,**HR max:** Heart rate maximum, **METs:** Metabolic equivalent of task, **Vo2 max :**The maximum amount of oxygen that an individual can utilise during intense or maximal exercise, **1 RM:** One repetition maximum, **CPET:** Cardiopulmonary exercise test, **HBCR:** Home based cardiac rehabilitation, **PA:** Physical activity, **CR:** Cardiac rehabilitation, **USA:** United States of America, **MVPA:** Moderate to vigorous intensity physical activity **AF:** Atrial fibrillation, **PCI:** Percutaneous coronary intervention, **TAVR**: Transcatheter aortic valve replacement, **Reps:** Repetitions, **CBCR:** Centre-based cardiac rehabilitation. |
| --- |

**Table A2: Data extraction and synthesis of exercise fidelity measurement**

| **Lead author (year)** | **Measure(s) of exercise fidelity** | **Adherence definition, description of the measure and the procedure of use** | **Reliability and validity of exercise fidelity measure(s)** | **Completion /dropout rates for fidelity measure(s)** | **Qualitative feedback /comments** **on feasibility /acceptability or limitations** |
| --- | --- | --- | --- | --- | --- |
| Aamot et al. (2016)  (1) | Self-reported adherence ((yes/no) to “exercising regularly” and to self-monitoring of exercise intensity | **Adherence definition:** Self-reporting of adherence to regular exercise (yes/no) and whether they used a (privately owned) heart rate monitor during exercise to guide exercise intensity.  **Procedure:**  Participants were instructed to wear heart rate monitors during exercise sessions to control their intensity. | Not reported | Not reported | Not reported |
| Batalik et al. (2018)  Batalik et al. (2020)  (2, 3) | A wearable device with (heart rate monitor, duration and distance) measurement | **Adherence definition**: The proportion of the 36 intended training sessions attended/undertaken during the home-based intervention.  **Procedure:**  b) As well as indicating “attendance”, the wearable device with heart rate monitors measured distance walked or cycled, duration of exercise, mode of exercise, time of exercise and heart rate.  b) The following were calculated from the data: Average heart rate reserve across all exercise sessions, the proportion of time spent on aerobic exercise, and time spent in the target heart rate zone**.**  b) A web-based app, *"polar flow ",* acted as a training diary. | Not reported | 5.4% of HR monitor data was lost due to battery issues. | Using a web application as a diary allowed physiotherapists and participants to access and review the training record.  a) Some patients (5%) had problems using the device due to low battery.  b) Some discontinued using the devices following battery issues.  b) Other technical problems (e.g., limited internet connection, difficulty using the software) were reported. |
| Bernocchi et al. (2016)(4) | 1. Exercise diaries  2. Borg RPE scale  3. Step count (pedometer) | **Adherence definition**: Performance of a minimum of 70% of prescribed training sessions.  **Procedure:**  a) Exercise diaries and Borg RPE completed at the end of each exercise session.  b) Progression of exercise was based on Borg RPE scale scoresand a review of step count, duration of exercise, oxygen saturation, HR, and blood pressure. | Not reported | Not applicable (protocol) | The study will provide data from questions on the device's usability. |
| Brouwers et al. (2017)  (5) | 1. Heart rate monitor  2.Accelerometer  3. Web application | **Adherence definition**: ≥ 50% of session time spent in the target heart rate zone (average per week) or ≥ 50% in the time spent in moderate to vigorous activity zone (average per day).  **Procedure:**  a) Accelerometers will be worn at the hip, along with heart monitors on wrists, throughout the intervention sessions.  b) A web application will review sensor data every four weeks and determine adherence using the above criteria. | Not reported | Not applicable (protocol) | Not reported |
| Cai et al. (2022)  (6) | 1. Wearable ECG  2. Mobile app | **Adherence definition:** The percentage of the 12 weeks during which the patient completed 150 or more minutes of exercise. Minutes spent at or above target HR were counted as exercise for each session.  **Procedure:**  a) Adherence was calculated from ECG records. Participants used the mobile app to upload data from daily training on HR average, HR mins, HR max, duration of exercise, and calories.  b) ECG monitored exercise HR so the participants could regulate the intensity during exercise.  c) ECG monitored safety issues such as palpation, chest pain, or arrhythmia, and participants transferred data to the centre. | Not reported | Not reported | Authors assume that HR and intensity monitoring will be acceptable to AF patients as after ablation they may feel less confident during exercise. |
| Claes et al. (2017)  (7) | 1. Heart rate monitor  2. Microsoft Kinect movement sensor  3. Blood pressure monitor  4. ECG | **Adherence definition**: Not defined  **Procedure:**  a) During each exercise session, the following data will be recorded:  1) Heart rate 2) METs 3) Movements  b) An avatar coach will give feedback on accuracy and intensity of movement during exercise sessions, guided by the *"Microsoft Kinect sensor"*.  c) Safety monitoring will be guided via a 3-lead ECG Sensor Device *(Intelesens)* along with wearing *"ActiGraph"* for 3 days.  e) Safety monitoring will include recording any muscle, tendon or ligament injuries. | Not reported | Not applicable (protocol) | Authors speculate that participants will be more likely to adhere to PATHway if they understand the system and like how it operates. |
| Ding et al. (2021)  (8) | A wearable activity device with a heart rate monitor and step counter (Philips Health Watch). | **Adherence definition**: The percentage of prescribed walking and training achieved. The number of minutes spent in the prescribed heart rate zone.  **Procedure:**  a) Daily step count measured throughout the study.  b) Progression or regression of exercises was based on completing pre-set goals. | Validation data for the watch is cited for heart rate and steps (Hendrikx et al., 2017). | Not reported | a) Participants wanted more support regarding technical issues with devices.  b) Participants wore study watches for a median of 61 (73%) of 84 study days. Average wear-time was 12.7 hours/day, decreasing by 0.6 hrs/week over 3 months. |
| Dor-Haim et al. (2019)  (9) | 1. A wearable ECG,12-lead (“Garment”)  2. Wrist band | **Adherence definition**: Not defined  **Procedure:**  a) Physiological data (heart rate minutes, blood pressure) were recorded via a smart watch and ECG garment.  b) Blood pressure was uploaded to an app by participants before and after the walking.  c) As a safety check, data on any abnormal alteration of heart rate or blood pressure, ability to achieve goals of the day and feelings of fatigue, frailty or palpitations were recorded on the app. | Not reported | Not applicable (protocol) | Data collection is only practical for those who are not digitally excluded. Authors believed that one major drawback of this method is the lack of synchronisation between the ECG and digital platform. This required patients and staff to switch between apps to match the data. |
| Dougherty et al. (2016)  (10) | 1. A wearable device with a Heart rate monitor  2. Step count (pedometer)  3. Exercise logbook  4.Borg Scale | **Adherence definition**: Performing at least 80% of prescribed exercise per week in terms of: training duration, numbers of walking times per day/week, steps, and training intensity (percentage of time spent in target heart rate zone).  **Procedure:**  a) A watch with a heart rate monitor was pre-programmed for the target HR for each participant. Data were uploaded every 2 weeks.  b) Participants recorded steps (from their pedometer), perceived exertion (Borg scale), duration of exercise and HR within 5 minutes following exercise in their logbooks. | Not reported | Not reported | a) Some faulty devices (which were replaced).  b) Authors reported some initial burden for participants from using heart rate monitors, logbooks, and pedometers. |
| Du et al. (2011)  (11) | 1.Exercise logbook  2.Lap counter | **Adherence definition**: Not defined  **Procedure:** Self-reporting of walk frequency, distance walked and duration in logbook (based on lap counter data and a timer). | Not reported | Not applicable (protocol) | The authors suggest that the use of self-reporting instruments may result in under or over-reporting. |
| Evangelista et al. (2005)  (12) | 1. Step count (pedometer)  2. Exercise logbook | **Adherence definition**: Not defined  **Procedure:** Participants recorded exercise, walking duration and step count (from pedometer), distance and the proportion of the prescribed number of walking minutes in a logbook. | The study found a significant correlation between functional outcome (Vo2 max, 6-minute walk test, ejection fraction) and measures of adherence. | 56% missing pedometer and exercise data in intervention arm. | The authors report that the pedometer was easy to understand and use. That it was relatively low cost (compared with accelerometers). However, The lack of a safety chain led to the loss of some pedometers and the authors speculate that financial incentives may have improved completion rates. |
| Giallauria et al. (2006)  (13) | 3. ECG Watch | **Adherence definition**: Not defined  **Procedure:**  a) Remote ECG monitoring.  b) ECG data were recorded at baseline and throughout exercise sessions. | Not reported | Not reported | Not reported |
| Higgins et al. (2001)  (14) | 1. Exercise logbook  2. RPE | **Adherence definition**: Not defined  **Procedure:**  a) Clinicians taught the participants how to score their RPE.  c) Participants recorded their RPE score, duration of walking, and frequency of walking on their exercise logs. | Not reported | Not reported | Not reported |
| Isakadze et al. (2024)  (15) | 1. Wearable watch  2. Blood pressure monitor | **Adherence definition:** Not defined  **Procedure:**  1) The app’s dashboard will show steps, HR, Blood pressure, exercise and physical activity.  2) The dashboard allows monitoring of any safety issues or red flags and asks participants to stop exercising if this happens. | Not reported | Not applicable (protocol) | The study will provide data on satisfaction and engagement with technology. |
| Karjalainen et al. (2012)  (16) | 1. Activity watch  2. Exercise logbook  3. HR recorder” | **Adherence definition**: Actual physical activity compared to mean training impulse (TRIMP). TRIMP was calculated as exercise time (minutes) x heart rate x *e*^1.92 x heart rate^ (for men) or *e*^1.67 x heart rate^ (for women).  **Procedure:** Participants used exercise diaries to record days with exercise, a predetermined heart rate zone, target exercise duration, and the mean heart rate (from the HR recorder) and exercise duration for each exercise session. | Not reported | Not reported | An advantage of using accelerometers as wristwatches in this study was that they made it easy for the participants to measure physical activity at any time of the day. However, some participants were not keen or unsure about using the wristwatches. |
| Korzeniowska-Kubacka et al. (2015)  (17) | ECG | **Adherence definition**: Not defined  **Procedure:**  a) Participants wore ECG devices which were pre-programmed to record intervals of training and duration during the session. ECG recordings were also done at the end of each training phase.  b) ECG monitored safety issues such as palpation, chest pain, or arrhythmia and confirmed whether exercise was within the prescribed heart rate zone. | Not reported | Not reported | Not reported |
| Li et al. (2019)  (18) | ECG | **Adherence definition**: This will be expressed as a rate based on adherence to the prescribed exercise. How the rate will be calculated was Not reported  **Procedure:**  ECG will be worn during the home-based exercise part of the intervention. | Not reported | Not applicable (protocol) | The authors suggest that:  1) The use of patients and public involvement for developing the intervention might enhance measurement (and intervention) adherence.  2) An advantage of ECG is allowing assessment of adherence to the prescribed intensity and alerting the patient (and doctor) of safety issues. |
| Li et al. (2015)  (19) | 1. step count (pedometer)  2. Exercise logbook  3. Borg RPE scale | **Adherence definition:** The proportion of weeks in which the participant reported exercising at least five times.  **Procedure:**  1) Participants were taught in the hospital to record their pulse rate for 10 seconds  Participants recorded the following in their exercise diaries:  Perceived exertion (RPE), duration of exercise, frequency of exercise, distance of walking, pulse rate, steps, completion of 14 exercises, Intolerance of any exercises and any symptoms or signs, and blood Pressure following exercise. | Not reported | Not reported | The authors suggest that the simple, safe and age specific exercises likely contributed to increased adherence. |
| Liu et al. (2022)  (20) | Exercise logbook | **Adherence definition:** > 75% of Tai Chi sessions attended.  **Procedure:**  1) Participants received exercise logbooks to record the daily duration and frequency of Tai Chi.  2) Participants received weekly phone calls to review logbook data, provide feedback and monitor compliance / to encourage intervention fidelity.  3) Participants received training on how to self-report intensity using the Borg scale. | Not reported | Not reported | Not reported |
| Lloyd et al. (2019)  (21) | 1.“Aerobic stepping” device with data capture (steps) linked to the internet  2. Borg RPE | **Adherence definition**: Indirectly defined as the slope of the magnitude of the exercise-time relationship. Also, the total number of minutes spent exercising on an "aerobic stepper" daily during 30 days of the intervention.  **Procedure:** Participants were asked to digitally record their duration of training at “aerobic steppers” per day into a *“Research Electronic Data Capture (REDCap)”* document for 30 days. | Not reported | Not reported | The authors pointed out that:  1) A common reason for the exclusion of interested participants was the lack of internet access. They hope to overcome this in the project's next phase via portable Wi-Fi devices and tablets.  2) Activity measurement was limited to the *"aerobic stepper"* only. They suggested that future studies should use multiple devices. |
| Nabutovsky et al. (2024)  (22) | 1. Wearable Smartwatch | **Adherence definition:** Not defined  **Procedure:** An online platform monitored intensity, steps, HR zone and usage of the app. | Not reported | Not reported | Not reported |
| Ngeno et al. (2022)  (23) | Step count (pedometer) | **Adherence definition:** The proportion of weekly step targets attained. CR was deemed feasible if at least 25% of sessions were completed.  **Procedure:**  1) Participants were instructed to wear pedometers at their hips.  2) Participants were contacted weekly to ask about step count.  4) The study trained participants to use the Borg scale and HR to assess exercise intensity. | Not reported | Not reported | a) Adherence was likely underreported as the pedometers malfunctioned. This may also have been influenced by the participants’ ability to choose home or centre CR.  b) The authors reported that using remote monitoring and mobile phone-based connectivity helped to minimise access barriers. |
| Nielsen et al. (2019)  (24) | 1.Exercise logbook  2.Participant rating on a scale  3. RPE | **Adherence definition**: Frequency of attendance of exercise sessions and adherence to the prescribed duration, intensity (RPE) and frequency of exercise.  **Procedure:** Participants were asked to fill out the exercise log. | Not reported | Not reported | The authors suggest that including patient choice to aid adherence needs to be explored further. |
| O’Connor et al. (2009) Jones et al. (2014)  (25, 26) | 1. Heart rate monitor  2. Activity log | **Adherence definition** No clear definition, but it was evaluated by measuring attendance at supervised training sessions, an activity logbook, telephone and clinic follow up and heart rate monitoring throughout home-based exercises.  **Procedure:** Participants were supplied with heart rate monitors and cycles to use at home. | Not reported | Not reported | Not reported |
| Oka et al. (2000)  Oka et al. (2005)  (27, 28) | 1-Activity log  2-Heart rate monitor  3-Borg RPE | **Adherence definition**: The proportion of prescribed weekly sessions that were attended. Exercise compliance was calculated every two weeks based on compliance with the prescribed aerobic and resistance training components of exercise.  **Procedure:**  a) Activity logbooks were used to compare weekly adherence to exercise targets. If participants did not return their logbook, they were contacted to obtain adherence data by phone.  b) Participants recorded symptoms, exercise time, RPE score, type of exercise, exercise frequency, exercise intensity and heart rate. | Not reported | Not reported | The authors suggest that the high adherence rates observed may be partly due to self-reporting bias. |
| Pater et al. (2000)  (29) | Activity-tracking wristwatch (brand not specified) | **Adherence definition**: Adherence (frequency of exercise) will be determined by the usage of a wristwatch throughout the intervention .  **Procedure:**  a) Intervention group will receive activity-tracker watches. No further details reported | Not reported | Not applicable (protocol) | Not reported |
| Piotrowicz et al. (2010)  Piotrowicz et al. (2014)  Piotrowicz et al. (2015)  (30-32) | 1.Exercise logbook  2. Attendance register  3.ECG  4.Borg RPE | **Adherence definition**: The percentage of the participants who completed >= 80% of both the prescribed cycling time and the prescribed number of exercise sessions.  Participants who achieved only one target (80% of prescribed cycling time or exercise sessions) were classified as partially non-compliant.  **Procedure:**  a) Data for ECG were transmitted to the call centre along with voice contact daily.  b) Safety of exercise was based on the following:  1-A subjective assessment of perceived exertion compared with the individualised exercise prescription.  2- Data from ECG.  3- Monitoring any contraindications of exercise training. | Not reported | Not reported | a) 5.3% of patients experienced skin reactions because of the electrodes. b) Participants felt safe with remote monitoring and supervision.  c) Survey results indicated that:  1) 1. 99% of participants found the device "very easy " to use.  2) 2.90% reported no issues with the device and its instructions.  3) Participants liked the daily communication with the centre.  4) Participants reported feeling safe doing unsupervised exercise at home. |
| Rawstorn et al. (2020)  (33) | 1.A Smartphone  2*.“A wearable sensor”* | **Adherence definition**: Not clear. However, they will monitor (at each exercise session) the participant's heart rate, respiratory rate, speed, location, distance, ECG and any cardiac symptoms.  **Procedure:**  **Stage 1(weeks 1 to 12):**  All the above data will be transmitted to the cloud using a web app. This will inform ongoing support, feedback, and exercise progression.  **Stage 2 (weeks 13 to 24):**  Participants will use wearable sensors and smartphone apps with no further monitoring or coaching by exercise physiologists. | Not reported | Not applicable (protocol) | A process evaluation including semi-instructed interviews will assess satisfaction, acceptability, engagement, and usability of the proposed intervention /adherence monitoring system. |
| Schmidt et al. (2024)  (34) | 1. Wearable Smartwatch (Heart rate monitor)  2. Exercise logbook  3. Phone call/interview | **Adherence definition:** The total number of exercise sessions attended. The smartwatch, telephone and exercise logs will be used to evaluate adherence.  **Procedure:**  1) Exercise sessions will be recorded using the watch and uploaded to an app, *“ Polar Flow”.*  2) Weekly phone call to monitor the participant's progress using HR data from the smartwatch, plus assessment of compliance with exercise prescription.  3) Participants will be advised to contact the rehab team if they feel any symptoms during or after training. | Not reported | Not applicable (protocol) | Not reported |
| Shen et al. (2024)  (35) | 1. Wearable watch (step counts, HR and other physical data )  2. Exercise logbook (app-based ) | **Adherence definition:** Not clearly defined  However, *“ the Exercise Adherence Rating Scale (EARS). ”* will be used at 1,3 and 6 months.  **Procedure:**  1) Participants will wear a watch to monitor step counts and HR during exercise.  2) An app will share the data with the medical team, participants and their families. If HR increases above a specified value, the app will notify the participants and their families for safety assurance.  3) Participants will use the app-based exercise log to record exercise intensity (Borg score) before and after exercise, watch usage, and any symptoms or signs before and after exercise, including BP and HR. Also, type of exercise, intensity, time and frequency /day or week. Any motivation to continue the exercise and any reason to stop exercise. | Not reported | Not applicable (protocol) | Not reported |
| Skobel et al. (2017)  (36) | 1.Portable ECG monitor  2.Exercise diary | **Adherence definition**: Not defined , although the number of exercise sessions completed was presented.  **Procedure:**  a) Data were collected via the ECG monitor.  b) Participants recorded the duration of exercise on their exercise diaries.  c) Patients' safety was checked before training sessions by asking about cardiac symptoms and signs. | Not reported | 20% of data were rejected due to technical issues.37 % of sessions were interrupted by the safety algorithms of the device. | Not reported |
| Smart et al. (2005)  (37) | 1.Heart rate monitor  2.Exercise logbook | **Adherence definition**: Number of hours at >60% heart rate max.  **Procedure:** The logbook was used to assess the number of hours the heart rate monitor was worn. Participants also recorded exercise duration, frequency, type, and perceived intensity. | Not reported | Not reported | Not reported |
| Suchy et al. (2014)  (38) | 1. Wearable heart rate sensor (chest strap)  2. Mobile application (to record HR)  3. Accelerometer | **Adherence definition**: The proportion of participants attending at least 70% of prescribed exercise sessions.  **Procedure:**  a) Participants will be instructed to wear the heart rate sensor during exercise.  b) If the adherence is below 70%, the site will contact the participant.  C) Participants will wear accelerometers for the whole programme to track their daily physical activity. | Not reported | Not applicable (protocol) | Not reported |
| Szalewska et al. (2015a)  (39) | Tele-ECG | **Adherence definition**: number of “dropouts” during the programme (which is the number of absentee days during the intervention).  **Procedure:** The parameters of the session prescription for each participant were pre-programmed individually for each participant in the ECG devices. After the session, ECG data was sent to the monitoring centre via the patients' phones. | Not reported | Not reported | Not reported |
| Szalewska et al. (2015b)  (40) | Tele-ECG | **Adherence definition**: The average proportion of non-attended days.  **Procedure:**  The study used a tele-ECG device to monitor the exercises in the home. Also, for supervision of exercise training. | Not reported | Not reported | Not reported |
| Torri et al. (2018)  (41) | Self-report via a web-based exercise diary | **Adherence definition**: The percentage of prescribed exercise time completed for a) aerobic and b) resistance exercise.  **Procedure:**  Participants recorded their daily physical activity, including the amount, time, and type on the web platform. The software converted the data to MET values. | Not reported | Not reported | 25% of ineligible participants had no internet or personal computer. |
| Varnfield et al. (2014)  (42) | 1. Smartphone-based diary  2. Accelerometer in phone | **Adherence definition**: Adherence to exercise was assessed via the uploaded data, but no specific definition or threshold for exercise adherence was provided.  **Procedure:** Accelerometer in the smartphone was used to collect intensity, duration and step count. | Not reported | Not reported | a) The authors reported that synchronising the smartphone with a web portal helped to collect data. b) Participants reported being motivated by activity monitoring and the associated app. |
| Vonk et al. (2021)  (43) | “Virtual training application “ | **Adherence definition:** Not clearly defined. Adherence will be reported as “descriptive statistics”.  **Procedure:**  1) The app will monitor compliance and check exercise intensity to see if any modification is needed. Weekly text messages will inform individualised progression.  2) Exercise feasibility, intensity and progression will be monitored weekly via the app.  3) Total exercise time and intensity will be increased throughout the intervention. | Not reported | Not applicable (protocol) | Not reported |
| Walters et al. (2010)  (44) | 1- Cell phone-based exercise diary  2-Accelerometer in phone | **Adherence definition**: Compliance with national physical activity guidelines will be assessed at baseline, six weeks, and six months follow-up via wearing a pedometer for seven days and lap with 6 minutes walk test.  **Procedure:**  a) Accelerometer in the smartphone will measure daily steps.  b) The *"Wellness Diary software"* will record important information including daily duration of exercise, heart rate and blood pressure. | Not reported | Not applicable (protocol) | Authors speculate that the technology used in this study may give the following advantages :  1-Accessible  2- Flexible  3-Cheap  4- Cost-effectiveness for providers compared with in-person intervention |
| Webb-Peploe et al. (2000)  (45) | 1. Exercise Diary.  2. Modified Borg scale | **Adherence definition**: Based on the numbers of the recorded revolutions pedalled and the XBX level performed. In addition, the number of days of exercising and completion of revolutions pedalled.  **Procedure:**  a) Participants were instructed to record in their exercise diaries the type and duration of their XBX exercise, exercise duration and the number of revolutions pedalled.  b) Participants were asked to end the exercise session if their heart rate was over 80% of the maximum heart rate achieved on an initial maximal treadmill test. It is not clear how the patient assessed HR. Also, the study instructed the participants to avoid any forms of exercise that caused difficulty of breathing or fatigue. | Not reported | Not reported | a) One major drawback was reported to be the lack of monitoring of exercise safety in terms of arrhythmia or symptoms /complications occurring during the intervention.  b) It was not possible to assess at what resistance the bicycle ergometry had been performed. |
| Xu et al. (2020)  (46) | 1.Self-reported “training log” uploaded via WeChat  2.Chest-worn heart rate monitor (type not specified)  3.*"smart insoles "* with plantar pressure sensors and accelerometers | **Adherence definition**: “Attendance” for 4 of the 6 weeks, based on the data from the *"Intelligent Exercise Rehabilitation Management System (IERMS)"*.  **Procedure:**  a) Heart rate monitors and smart insoles will record heart rate, intensity, and type of exercise during each exercise session.  b) A mobile app will automatically guide patients during exercise to ensure they work within a predetermined zone.  c) Participants will be assessed (via the app) for safety before the session, including heart rate, blood pressure, and any cardiac signs or symptoms (e.g., chest distress or dyspnoea) and informed if they are safe to exercise. | The paper cited Item-Glatthorn et al. (2012) which found that the device used *was a* valid measure of gait, by comparison to a reference method in patients with hip osteoarthritis(47). | Not applicable (protocol) | The authors state that one drawback of their approach is that only participants with smartphone and internet will be able to use the devices. |

| **CAD:** Coronary artery disease**, MI:** Myocardial infarction**: CVD:** Cardiovascular disease**, COPD:** Chronic obstructive pulmonary disease**, CHF:** Chronic heart failure**, HF:** Heart failure **,IHD:** Ischaemic heart disease**, ICD:** Implantable cardioverter defibrillator**, PAD:** Peripheral arterial disease**, AMI:** Acute myocardial infarction**, PCI:** Percutaneous coronary intervention**, HFrEF:** Heart failure with a reduced ejection fraction, **CABAG:** A coronary Artery Bypass Graft**, PTCA:** Percutaneous transluminal coronary angioplasty**, ACS:** Acute coronary syndrome**, CHD:** Coronary heart disease **,EF:** Ejection fraction**, LVEF:** Left Ventricular ejection fraction**, HFpEF:** Heart failure with preserved ejection fraction**, NSTEMI:** Non-ST segment elevation myocardial infarction**, STEMI:** ST-Segment Elevation Myocardial Infarction**, RCT**: Randomised controlled trial, **CR:** Cardiac rehabilitation**, HIIT** : High-intensity interval training**, HRR:** Heart rate reserve **,RPE:** Rated Perceived Exertion **HR**: Heart rate, **HR max :**Heart rate maximum**, METs:** Metabolic equivalent of task,**Vo2 max**: The maximum amount of oxygen that an individual can utilise during intense or maximal exercise**, 1RM:** One repetition maximum, **PA:** Physical activity**, CR:** Cardiac rehabilitation**, USA**: United States of America, **MVPA:** Moderate to vigorous intensity physical activity **AF:** Atrial fibrillation, **PCI:** Percutaneous coronary intervention, **TAVR**: Transcatheter aortic valve replacement, **Reps:** Repetitions, **CBCR:** Centre-based cardiac rehabilitation. |
| --- |

**(List of references)**

1. Aamot IL, Karlsen T, Dalen H, Stoylen A. Long-term Exercise Adherence After High-intensity Interval Training in Cardiac Rehabilitation: A Randomized Study. Physiother Res Int. 2016;21(1):54-64.

2. Batalik L, Dosbaba F, Hartman M, Batalikova K, Spinar J. Rationale and design of randomized controlled trial protocol of cardiovascular rehabilitation based on the use of telemedicine technology in the Czech Republic (CR-GPS). Medicine (United States). 2018;97(37).

3. Batalik L, Dosbaba F, Hartman M, Batalikova K, Spinar J. Benefits and effectiveness of using a wrist heart rate monitor as a telerehabilitation device in cardiac patients: A randomized controlled trial. Medicine (Baltimore). 2020;99(11):e19556.

4. Bernocchi P, Scalvini S, Galli T, Paneroni M, Baratti D, Turla O, et al. A multidisciplinary telehealth program in patients with combined chronic obstructive pulmonary disease and chronic heart failure: study protocol for a randomized controlled trial. Trials. 2016;17(1):462.

5. Brouwers RW, Kraal JJ, Traa SC, Spee RF, Oostveen LM, Kemps HM. Effects of cardiac telerehabilitation in patients with coronary artery disease using a personalised patient-centred web application: protocol for the SmartCare-CAD randomised controlled trial. BMC Cardiovasc Disord. 2017;17(1):46.

6. Cai C, Bao Z, Wu N, Wu F, Sun G, Yang G, Chen M. A novel model of home-based, patient-tailored and mobile application-guided cardiac telerehabilitation in patients with atrial fibrillation: A randomised controlled trial. Clinical Rehabilitation. 2022;36(1):40-50.

7. Claes J, Buys R, Woods C, Briggs A, Geue C, Aitken M, et al. PATHway I: design and rationale for the investigation of the feasibility, clinical effectiveness and cost-effectiveness of a technology-enabled cardiac rehabilitation platform. BMJ Open. 2017;7(6):e016781.

8. Ding EY, Erskine N, Stut W, McManus DD, Peterson A, Wang Z, et al. MI-PACE Home-Based Cardiac Telerehabilitation Program for Heart Attack Survivors: Usability Study. JMIR Hum Factors. 2021;8(3):e18130.

9. Dor-Haim H, Katzburg S, Leibowitz D. A Novel Digital Platform for a Monitored Home-based Cardiac Rehabilitation Program. J Vis Exp. 2019(146).

10. Dougherty CM, Luttrell MN, Burr RL, Kim M, Haskell WL. Adherence to an Aerobic Exercise Intervention after an Implantable Cardioverter Defibrillator (ICD). Pacing Clin Electrophysiol. 2016;39(2):128-39.

11. Du HY, Newton PJ, Zecchin R, Denniss R, Salamonson Y, Everett B, et al. An intervention to promote physical activity and self-management in people with stable chronic heart failure The Home-Heart-Walk study: study protocol for a randomized controlled trial. Trials. 2011;12(1):63.

12. Evangelista LS, Dracup K, Erickson V, McCarthy WJ, Hamilton MA, Fonarow GC. Validity of pedometers for measuring exercise adherence in heart failure patients. J Card Fail. 2005;11(5):366-71.

13. Giallauria F, Lucci R, Pilerci F, De Lorenzo A, Manakos A, Psaroudaki M, et al. Efficacy of telecardiology in improving the results of cardiac rehabilitation after acute myocardial infarction. Monaldi Arch Chest Dis. 2006;66(1):8-12.

14. Higgins HC, Hayes RL, McKenna KT. Rehabilitation outcomes following percutaneous coronary interventions (PCI). Patient Educ Couns. 2001;43(3):219-30.

15. Isakadze N, Kim CH, Marvel FA, Ding J, MacFarlane Z, Gao Y, et al. Rationale and Design of the mTECH‐Rehab Randomized Controlled Trial: Impact of a Mobile Technology Enabled Corrie Cardiac Rehabilitation Program on Functional Status and Cardiovascular Health. Journal of the American Heart Association. 2024;13(2):e030654.

16. Karjalainen JJ, Kiviniemi AM, Hautala AJ, Niva J, Lepojarvi S, Makikallio TH, et al. Effects of exercise prescription on daily physical activity and maximal exercise capacity in coronary artery disease patients with and without type 2 diabetes. Clinical Physiology and Functional Imaging. 2012;32(6):445-54.

17. Korzeniowska-Kubacka I, Bilinska M, Dobraszkiewicz-Wasilewska B, Piotrowicz R. Hybrid model of cardiac rehabilitation in men and women after myocardial infarction. Cardiol J. 2015;22(2):212-8.

18. Li J, Yang P, Fu D, Ye X, Zhang L, Chen G, et al. Effects of home-based cardiac exercise rehabilitation with remote electrocardiogram monitoring in patients with chronic heart failure: a study protocol for a randomised controlled trial. BMJ Open. 2019;9(3):e023923.

19. Li X, Xu S, Zhou L, Li R, Wang J. Home-Based Exercise in Older Adults Recently Discharged From the Hospital for Cardiovascular Disease in China: Randomized Clinical Trial. Nurs Res. 2015;64(4):246-55.

20. Liu T, Chan AWK, Chair SY. Group-plus home-based Tai Chi program improves functional health among patients with coronary heart disease: a randomized controlled trial. European Journal of Cardiovascular Nursing. 2022;21(6):597-611.

21. Lloyd T, Buck H, Foy A, Black S, Pinter A, Pogash R, et al. The Penn State Heart Assistant: A pilot study of a web-based intervention to improve self-care of heart failure patients. Health Informatics J. 2019;25(2):292-303.

22. Nabutovsky I, Breitner D, Heller A, Levine Y, Moreno M, Scheinowitz M, et al. Home-based cardiac rehabilitation among patients unwilling to participate in hospital-based programs. Journal of Cardiopulmonary Rehabilitation and Prevention. 2024;44(1):33-9.

23. Ngeno GTK, Barasa F, Kamano J, Kwobah E, Wambui C, Binanay C, et al. Feasibility of Cardiac Rehabilitation Models in Kenya. Annals of Global Health. 2022;88(1).

24. Nielsen J, Duncan K, Pozehl B. Patient-Selected Strategies for Post Cardiac Rehabilitation Exercise Adherence in Heart Failure. Rehabil Nurs. 2019;44(3):181-5.

25. O'Connor CM, Whellan DJ, Lee KL, Keteyian SJ, Cooper LS, Ellis SJ, et al. Efficacy and safety of exercise training in patients with chronic heart failure: HF-ACTION randomized controlled trial. JAMA. 2009;301(14):1439-50.

26. Jones LW, Douglas PS, Khouri MG, Mackey JR, Wojdyla D, Kraus WE, et al. Safety and efficacy of aerobic training in patients with cancer who have heart failure: an analysis of the HF-ACTION randomized trial. J Clin Oncol. 2014;32(23):2496-502.

27. Oka RK, De Marco T, Haskell WL, Botvinick E, Dae MW, Bolen K, Chatterjee K. Impact of a home-based walking and resistance training program on quality of life in patients with heart failure. Am J Cardiol. 2000;85(3):365-9.

28. Oka RK, DeMarco T, Haskell WL. Effect of treadmill testing and exercise training on self-efficacy in patients with heart failure. Eur J Cardiovasc Nurs. 2005;4(3):215-9.

29. Pater C, Ditlef Jacobsen C, Rollag A, Sandvik L, Erikssen J, Karin Kogstad E. Design of a randomized controlled trial of comprehensive rehabilitation in patients with myocardial infarction, stabilized acute coronary syndrome, percutaneous transluminal coronary angioplasty or coronary artery bypass grafting: Akershus Comprehensive Cardiac Rehabilitation Trial (the CORE Study). Curr Control Trials Cardiovasc Med. 2000;1(3):177-83.

30. Piotrowicz E, Baranowski R, Bilinska M, Stepnowska M, Piotrowska M, Wojcik A, et al. A new model of home-based telemonitored cardiac rehabilitation in patients with heart failure: effectiveness, quality of life, and adherence. Eur J Heart Fail. 2010;12(2):164-71.

31. Piotrowicz E, Korzeniowska-Kubacka I, Chrapowicka A, Wolszakiewicz J, Dobraszkiewicz-Wasilewska B, Batogowski M, et al. Feasibility of home-based cardiac telerehabilitation: Results of TeleInterMed study. Cardiol J. 2014;21(5):539-46.

32. Piotrowicz E, Zielinski T, Bodalski R, Rywik T, Dobraszkiewicz-Wasilewska B, Sobieszczanska-Malek M, et al. Home-based telemonitored Nordic walking training is well accepted, safe, effective and has high adherence among heart failure patients, including those with cardiovascular implantable electronic devices: a randomised controlled study. Eur J Prev Cardiol. 2015;22(11):1368-77.

33. Rawstorn JC, Ball K, Oldenburg B, Chow CK, McNaughton SA, Lamb KE, et al. Smartphone Cardiac Rehabilitation, Assisted Self-Management Versus Usual Care: Protocol for a Multicenter Randomized Controlled Trial to Compare Effects and Costs Among People With Coronary Heart Disease. JMIR Res Protoc. 2020;9(1):e15022.

34. Schmidt C, Magalhães S, Basilio PG, Gouveia M, Teixeira M, Santos C, et al. Home-versus centre-based EXercise InTervention in patients with Heart Failure (EXIT-HF trial): A pragmatic randomized controlled trial. Revista Portuguesa de Cardiologia. 2024;43(3):149-58.

35. Shen Z, Mi S, Huang C, Zhou D, Pan W, Xu X, et al. Home-based mobile-guided exercise-based cardiac rehabilitation among patients undergoing transcatheter aortic valve replacement (REHAB-TAVR): protocol for a randomised clinical trial. BMJ open. 2024;14(3):e080042.

36. Skobel E, Knackstedt C, Martinez-Romero A, Salvi D, Vera-Munoz C, Napp A, et al. Internet-based training of coronary artery patients: the Heart Cycle Trial. Heart Vessels. 2017;32(4):408-18.

37. Smart N, Haluska B, Jeffriess L, Marwick TH. Predictors of a sustained response to exercise training in patients with chronic heart failure: a telemonitoring study. Am Heart J. 2005;150(6):1240-7.

38. Suchy C, Massen L, Rognmo O, Van Craenenbroeck EM, Beckers P, Kraigher-Krainer E, et al. Optimising exercise training in prevention and treatment of diastolic heart failure (OptimEx-CLIN): rationale and design of a prospective, randomised, controlled trial. Eur J Prev Cardiol. 2014;21(2 Suppl):18-25.

39. Szalewska D, Niedoszytko P, Gierat-Haponiuk K. The impact of professional status on the effects of and adherence to the outpatient followed by home-based telemonitored cardiac rehabilitation in patients referred by a social insurance institution. Int J Occup Med Environ Health. 2015;28(4):761-70.

40. Szalewska D, Tomaszewski J, Kusiak-Kaczmarek M, Niedoszytko P, Gierat-Haponiuk K, Haponiuk I, Bakuła S. Influence of a hybrid form of cardiac rehabilitation on exercise tolerance in coronary artery disease patients with and without diabetes. Kardiol Pol. 2015;73(9):753-60.

41. Torri A, Panzarino C, Scaglione A, Modica M, Bordoni B, Redaelli R, et al. Promotion of Home-Based Exercise Training as Secondary Prevention of Coronary Heart Disease: A Pilot Web-Based Intervention. Journal of Cardiopulmonary Rehabilitation and Prevention. 2018;38(4):253-8.

42. Varnfield M, Karunanithi M, Lee CK, Honeyman E, Arnold D, Ding H, et al. Smartphone-based home care model improved use of cardiac rehabilitation in postmyocardial infarction patients: results from a randomised controlled trial. Heart. 2014;100(22):1770-9.

43. Vonk T, Bakker EA, Zegers ES, Hopman MT, Eijsvogels TM. Effect of a personalised mHealth home-based training application on physical activity levels during and after centre-based cardiac rehabilitation: rationale and design of the Cardiac RehApp randomised control trial. BMJ Open Sport & Exercise Medicine. 2021;7(3):e001159.

44. Walters DL, Sarela A, Fairfull A, Neighbour K, Cowen C, Stephens B, et al. A mobile phone-based care model for outpatient cardiac rehabilitation: the care assessment platform (CAP). BMC Cardiovasc Disord. 2010;10:5.

45. Webb-Peploe KM, Chua TP, Harrington D, Henein MY, Gibson DG, Coats AJ. Different response of patients with idiopathic and ischaemic dilated cardiomyopathy to exercise training. Int J Cardiol. 2000;74(2-3):215-24.

46. Xu L, Xiong W, Li J, Shi H, Shen M, Zhang X, et al. Role of the intelligent exercise rehabilitation management system on adherence of cardiac rehabilitation in patients with coronary heart disease: a randomised controlled crossover study protocol. BMJ Open. 2020;10(6):e036720.

47. Item-Glatthorn JF, Casartelli NC, Petrich-Munzinger J, Munzinger UK, Maffiuletti NA. Validity of the intelligent device for energy expenditure and activity accelerometry system for quantitative gait analysis in patients with hip osteoarthritis. Archives of physical medicine and rehabilitation. 2012;93(11):2090-3.
